# Dissociated Primary Human Prostate Cancer Cells Coinjected with the Immortalized Hs5 Bone Marrow Stromal Cells Generate Undifferentiated Tumors in NOD/SCID-γ Mice

**DOI:** 10.1371/journal.pone.0056903

**Published:** 2013-02-22

**Authors:** Xin Chen, Bigang Liu, Qiuhui Li, Sofia Honorio, Xin Liu, Can Liu, Asha S. Multani, Tammy Calhoun-Davis, Dean G. Tang

**Affiliations:** 1 Department of Molecular Carcinogenesis, The University of Texas M.D. Anderson Cancer Center, Science Park, Smithville, Texas, United States of America; 2 Program in Molecular Carcinogenesis, Graduate School of Biomedical Sciences (GSBS), Houston, Texas, United States of America; 3 Center of Stomatology, Tongji Hospital, Tongji Medical College, Huazhong University of Science and Technology, Wuhan, China; 4 Department of Nutritional Science, University of Texas at Austin, Austin, Texas, United States of America; 5 Department of Genetics, the University of Texas M.D. Anderson Cancer Center, Houston, Texas, United States of America; 6 Centers for Cancer Epigenetics, Stem Cell and Developmental Biology, RNA Interference and Non-coding RNAs, and Molecular Carcinogenesis, University of Texas M.D. Anderson Cancer Center, Houston, Texas, United States of America; 7 Cancer Stem Cell Institute, Research Center for Translational Medicine, East Hospital, Tongji University School of Medicine, Shanghai, China; University of Dayton, United States of America

## Abstract

Reconstitution of tumor development in immunodeficient mice from disaggregated primary human tumor cells is always challenging. The main goal of the present study is to establish a reliable assay system that would allow us to reproducibly reconstitute human prostate tumor regeneration in mice using patient tumor-derived single cells. Using many of the 114 untreated primary human prostate cancer (HPCa) samples we have worked on, here we show that: 1) the subcutaneum represents the most sensitive site that allows the grafting of the implanted HPCa pieces; 2) primary HPCa cells by themselves fail to regenerate tumors in immunodeficient hosts; 3) when coinjected in Matrigel with rUGM (rat urogenital sinus mesenchyme), CAF (carcinoma-associated fibroblasts), or Hs5 (immortalized bone marrow derived stromal) cells, primary HPCa cells fail to initiate serially transplantable tumors in NOD/SCID mice; and 4) however, HPCa cells coinjected with the Hs5 cells into more immunodeficient NOD/SCID-IL2Rγ^−/−^ (NSG) mice readily regenerate serially transplantable tumors. The HPCa/Hs5 reconstituted ‘prostate’ tumors present an overall epithelial morphology, are of the human origin, and contain cells positive for AR, CK8, and racemase. Cytogenetic analysis provides further evidence for the presence of karyotypically abnormal HPCa cells in the HPCa/Hs5 tumors. Of importance, HPCa/Hs5 xenograft tumors contain EpCAM^+^ cells that are both clonogenic and tumorigenic. Surprisingly, all HPCa/Hs5 reconstituted tumors are undifferentiated, even for HPCa cells derived from Gleason 7 tumors. Our results indicate that primary HPCa cells coinjected with the immortalized Hs5 stromal cells generate undifferentiated tumors in NSG mice and we provide evidence that undifferentiated HPCa cells might be *the* cells that possessed tumorigenic potential and regenerated HPCa/Hs5 xenograft tumors.

## Introduction

Prostate cancer (PCa) is the leading malignancy with estimated ∼241,740 new cases and ∼ 28,170 deaths in the USA in 2012 [1]. The etiology for PCa remains enigmatic and the cells-of-origin for castration-resistant PCa (i.e., CRPC), the lethal disease that kills most patients remains poorly defined. Human cancers harbor a population of stem-like cancer cells operationally termed cancer stem cells (CSCs), which are believed to be responsible for tumor initiation, promotion, progression, metastasis, and treatment resistance [2]. Work from our lab and many others’ suggests that human PCa also contains stem-like cancer cells [3]–[32]. Like CSCs in other tumors [33], prostate CSCs are heterogeneous containing many subsets with distinct tumor-regenerating capacity. Of note, prostate CSCs reported by several groups are less differentiated expressing little/no AR (androgen receptor) and PSA (prostate-specific antigen). Recently, using a PSA promoter-driven GFP lentiviral reporter, we have purified out differentiated (PSA^+^) and undifferentiated (PSA^−/lo^) PCa cells for gene expression profiling and functional studies and found that the PSA^−/lo^ cell population harbors long-term tumor-propagating cells that resist to castration [25]. Our study suggests that the undifferentiated PSA^−/lo^ PCa cell population likely represents a pre-existent cell-of-origin for CRPC [25].

A KEY unanswered question is whether similar stem-like PCa cells with enhanced tumor-propagating properties also exist in primary human PCa (HPCa) samples. The reason that this important question has dodged a definitive answer lies in the fact that we have yet to establish a RELIABLE assay system that can REPRODUCIBLY and FAITHFULLY reconstitute tumor regeneration from dissociated HPCa single cells [14]. Most currently used PCa models are derived from either genetically modified mice where specific genes are overexpressed or knocked out or from xenografts by using human cancer cell lines or tumor pieces inoculated orthotopically or ectopically into the immunodeficient mice [34]. For many reasons, mouse models of PCa possess histopathological characteristics that are not entirely representative of human PCa, which are often characterized by multiple genetic alterations that are beyond the ability of any genetically engineered models may recapitulate. Moreover, a specific genetic mutation may result in distinct biological and histological phenotypes in animals versus in human [35]. In contrast, xenograft models are widely studied for the ease of use. They are of human origins and therefore are believed to better recapitulate human tumors in terms of the histopathological and molecular characteristics [34].

Several widely used PCa xenografts, such as the LAPC and LuCaP series [36]–[38], have been established by implanting human prostate tumor pieces in mice. PCa xenografts can also be created by injecting established PCa cell lines such as PC3, Du145, and LNCaP [39]. Due to the well-known fact that localized PCa or PCa cells rarely form tumors in immunodeficient mice [39], the above-mentioned examples of xenografts or cell lines were all established from metastases, and they only represent a minority of surgically removed human PCa and do not completely reflect the heterogeneity of the disease [40]. Recently, efforts have been made to generate PCa xenografts by grafting localized PCa pieces [41], [42] or primary PCa cells recombined with neonatal mouse mesenchyme [43] in the renal capsule. The regenerated xenografts appear to resemble, histopathologically, the donor patient tumors, but whether they could be serially passaged is unknown.

The main goal of our current project is to establish a reliable assay system that would allow us to reproducibly and faithfully reconstitute human prostate tumor regeneration in mice using patient tumor-derived HPCa single cells. We have previously made some efforts towards this goal but most of our reconstitution protocols completely failed to regenerate tumors [14]. Here, we have utilized many of the 114 untreated prostatectomy samples, ranging from Gleason score (GS) 6 to 10, to prepare single epithelial cancer cells, which were then recombined with different stromal cells including rUGM, carcinoma-associated fibroblasts (CAFs), or immortalized bone marrow-derived stromal cells (Hs5), and implanted at different anatomical sites in either NOD/SCID or NOD/SCID-IL2Rγ^−/−^ (NSG) mice. Below we present the results of our comprehensive studies.

## Materials and Methods

All animal-related studies in this project have been approved by the M.D. Anderson Cancer Center IACUC (Institutional Animal Care and Use Committee; ACUF# 08-05-08132). The current research does not involve human subjects (i.e., living individuals or identifiable private information) although it does involve primary HPCa samples obtained from our collaborating clinicians under the coverage of IRB (Institutional Review Board) Protocol number LAB04-0498. All other studies presented herein were the investigator-initiated and did not require approval from other regulatory bodies.

### Cells, Reagents, and Animals

PC3, DU145, LNCaP, and Swiss 3T3 cells were obtained from ATCC. The Hs5 cell line, which was generated from human bone marrow and immortalized by transduction with human papilloma virus E6/7 genes [44], was kindly provided by Dr. M. Andreeff (M.D Anderson Cancer Center). Carcinoma associated fibroblasts (CAFs) were prepared as previously reported [45]. All cells were cultured in recommended media containing 7% heat-inactivated FBS, 100 µg/ml streptomycin, and 200 U/ml penicillin (Gibco). Testosterone was purchased from Sigma. The TRPC xenograft line was provided by Dr. Palapattu [46]. Immunodeficient mice (NOD/SCID, NSG, Rag2; see ref. 14 about the properties of these animal strains) were initially purchased from the Jackson Laboratories (Bar Harbor, ME) and the breeding colonies were established in our animal facility and maintained in standard conditions according to the institutional guidelines. Antibodies used in this study are presented in Table S1.

### Histological and Immunohistochemical (IHC) Analyses

Tumor tissues harvested from patient tumors and reconstituted xenografts were fixed in 10% neutral buffered formalin for 24 h followed by 70% ethanol and embedding in paraffin. Sections (4 µm) were cut and stained with hematoxylin and eosin (HE). For IHC, sections were deparaffinized and hydrated and endogenous peroxidase activity was blocked with 3% H_2_O_2_ in water for 10 min. Antigen retrieval was performed with 10 mM citrate buffer (pH 6.0) for 10 min in a microwave oven followed by a 20-min cool down and thorough wash. Slides were incubated with Biocare Blocking Reagent (#BS966M with casein in the buffer) for 10 min to block non-specific binding. Slides were incubated with various primary antibodies for 30 min at room temperature, and washed in phosphate buffer twice and then incubated in biotinylated goat-anti-rabbit or mouse IgG (Vector Laboratories, Burlingame, CA) at a 1∶500 dilution for 30 min at room temperature. After thorough washing, they were incubated with SA-HRP (BioGenex, San Ramon, CA) for 30 min at room temperature followed by washing. Finally, these slides were incubated with BioGenex DAB substrate (color development closely monitored under a microscope) and lightly counterstained with hematoxylin. Images were captured using a MagnaFire Camera, and the whole mount slides were scanned using an Aperio ImageScope system.

### Aperio-assisted Morphometric Analysis

HE or IHC stained glass slides containing patient or xenograft tumors were scanned by using the Aperio ScanScope imaging platform (Aperio Technologies, Vista, CA, USA) with a 20× objective at a spatial sampling period of 0.47 *µ*m per pixel. Whole slides images were viewed and analyzed by using desktop personal computers equipped with the free ScanScope software.

### Purification of Tumor Cells from Primary Patient Samples and Xenograft Tumors

Basic procedures have recently been described [14]. Primary PCa samples were obtained at radical prostatectomy with patients’ consent according to the MDACC Institutional Review Board guidelines (IRB LAB04-0498). None of the patients received any treatment prior to surgery. *For patient samples*, tumor tissues freshly obtained from prostatectomy were minced into ∼1 mm^3^ pieces and tissues are subjected to enzymatic digestion (type I collagenase plus DNase at 50 U/ml) for 8–10 h at 37°C. Upon digestion, epithelial organoids are enriched by a brief centrifugation followed by trypsin digestion (0.05%, Gibco) on a rocker at 37°C for 15–30 min to release epithelial cells. *For xenografts*, tumor tissues were incubated with 1× Accumax (1200–2000 U/ml proteolytic activity containing collagenase and DNase; Innovative Cell Technologies, Inc) at 10 ml per gram tissue for 30 min at room temperature under rotating conditions. Single-cell suspension was obtained by filtering the supernatant through a pre-wetted 40-µm cell strainer and cell suspension was then gently loaded onto a layer of Histopaque-1077 (Sigma) gradient purification step to remove the majority of red blood cells, dead cells and debris. Finally, the resultant cell mixture was subjected to a MACS lineage cell depletion kit (Miltenyi Biotec) and the cocktail (anti-CD3, 14, 16, 19, 20, 45, 56, and 140b for patient tumors; H2K^d^ for xenografts) to remove the Lin^+^ cells including hematopoietic, endothelial, and other stromal cells (smooth muscle, myoepithelial, fibroblast, etc) for patient samples, or mouse stromal cells for xenograft tumors, respectively.

### Tumor Transplantation Experiments

Purified PCa cells (above), either alone or in combination with helper cells including rUGM, CAFs, or Hs5 cells, are implanted either subcutaneously (s.c) or under the kidney capsule (KC) of immuno-deficient mice. Alternatively, pieces or fragments of HPCa were grafted subcutaneously, under the KC, or in the mouse anterior prostate (AP). Basic procedures for these transplantations have been previously described [14]. Briefly, *for s.c implantations*, tumor cells were injected, in 40 µl of medium containing 50% Matrigel, subcutaneously into 6–8 week old male mice supplemented with exogenous testosterone. *For KC transplantations*, PCa cells were mixed with 250,000 rUGM cells and tissue recombinants were made in rat-tail collagen and incubated overnight. The tissue recombinants were transplanted next morning under the renal capsule of recipient male mice supplemented with testosterone pellets. *For AP grafting*, small pieces of HPCa tissues were directly implanted. In some cases, HPCa cells at different numbers were first mixed with rat collagen and incubated in a tissue culture plate at 37°C for 10–15 min. Then the solidified cell pellets were gently covered in medium and cultured for 4 h to overnight prior to implantation. A transverse incision was made in the lower abdomen to expose the AP by partially pulling the bladder, seminal vesicles and prostate out of the abdominal cavity. A 2–3 mm incision was made in the AP through the tubule between the two main ducts with the aid of a 22-gauge needle. Using a fire-rounded glass pipette tip, the collagen dots were inserted into a pocket formed under the prostate tubule. Then the organs were replaced and the body wall and skin closed.

In all above experiments, tumor development was monitored starting from the second week. Tumorigenicity was measured mainly by tumor incidence (i.e., the number of tumors/number of injections), latency (i.e., time from injection to detection of palpable tumors), tumor volume, and endpoint tumor weight.

### Western Blotting

Whole cell lysates were prepared in complete RIPA buffer (50 mM Tris-HCl, pH 7.5, 150 mM NaCl, 1% Nonidet P-40, 0.5% sodium deoxycholate, 0.5% Triton X-100, 10 mM EDTA) containing protease inhibitor mixture. Protein concentrations were determined by MicroBCA kit (Pierce). Various amounts of proteins were loaded on a 15% SDS-PAGE. Western blotting was performed as standard using ECL Plus (PerkinElmer).

### Reverse Transcription (RT)-PCR Analysis

Total RNA was extracted from tumor pieces or cultured cancer cells using Trizol (Invitrogen), and used in RT-PCR analysis. The PCR primers included human AR (sense: 5′-GCTAAAGACTCGGAGGAAGCAAG-3′; antisense: 5′-TGGGGGAAAACAGAGGGTTC-3′); PSA (sense: 5′-TGGGAGTGCGAGAAGCATTC-3′; antisense: 5′-GCTGTGGCTGACCTGAAATACC-3′); β-actin (sense:5′-CTGGCACCACACCTTCTACAATG-3′; antisense: 5′- AATGTCACGCACGATTTCCCGC-3′).

### Karyotyping and Genomic Instability

Cells were exposed to Colcemid (0.04 *µ*g/ml) for 25 min at 37°C and then to a hypotonic solution (0.075 M KCl) for 20 min at room temperature. Cells were fixed in a methanol and acetic acid (3∶1 by volume) mixture for 15 min, and washed three times in the fixative. The slides were air-dried, optimally aged, and G-banded using trypsin solution and stained in Giemsa following the routine procedure. Images were captured using a Nikon 80i microscope equipped with karyotyping software from Applied Spectral Imaging (ASI) Inc. (Vista, CA). and a minimum of 15 G-banded metaphases were karyotyped.

### Fluorescence-Activated Cell Sorting (FACS) and Purification of EpCAM^+^ Cells

HPCa/Hs5 xenograft tumors cells were treated with FcR blocking agent (Miltenyi Biotec) for 10–15 min at 4°C and stained with PerCP-eFluor 710 conjugated anti-EpCAM antibody and biotinylated mouse H-2K^d^ antibody for 30 min at 4°C. Cells were then washed with PBS and labeled with Alexa Fluor 405 conjugated streptavidin (Invitrogen, S-32351) for 10 min at 4°C. EpCAM^+^ and EpCAM^−^ cells were further purified using FACS [21], [25]. Post-analysis revealed purities of both populations being >98%.

### Sphere Formation Assays

Basic procedures for sphere formation assays were previously described [21], [25]. We purified EpCAM^+^ cells from the HPCa/Hs5 xenograft tumors via MACS, and cultured them up in serum-free prostate epithelial basal medium (PrEBM) containing B27 (Invitrogen), 20 ng/ml EGF and bFGF in Purecol (Advanced BioMatrix, #5005-B) coated T-25 flasks. When these cells were confluent, we harvested the cells via 0.05% trypsin/EDTA, and plated the single cells in 6-well ULA plates at the density of 5,000–10,000 cells/well. Spheres were scored in ∼2 weeks. For serial sphere-formation assays, the first-generation spheres were harvested with 0.025% trypsin/EDTA, triturated with a 27-G needle, filtered through a 40-µm strainer, and replated as above. This process was repeated for up to 3–4 generations.

### Statistic Analysis

Tumor-initiating cell frequencies were compared using likelihood ratio tests. Differences in tumor-take rate were determined by the Proportion test. Statistical significance was defined as *P*<0.05.

## Results

### HPCa Piece Xenotransplants Show Higher Tumor Takes at Subcutaneous Site than at Other Sites

**Figure 1:**
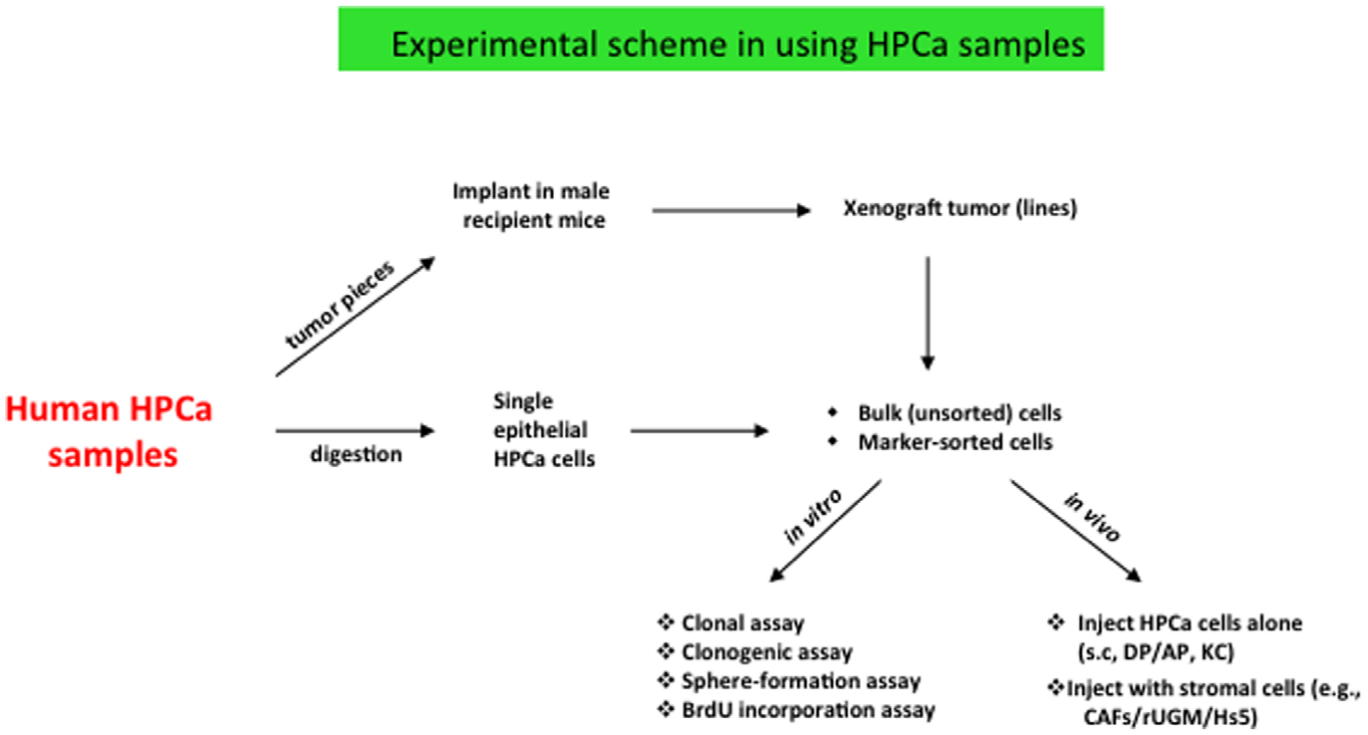
Experimental scheme in using HPCa samples. See Text for description.

Our lab has so far worked on 114 patient samples freshly obtained from prostatectomy. Some of these samples have been used in our previous studies [13], [14], [21], [22], [25], [31] and most of them were used in the current project (Table S2). Of the 114 HPCa samples, 15.8% had combined Gleason Score (GS) 6, 52.6% GS7, 11.4% GS8, and 20.2% GS9. In general, the HPCa samples were used in 2 types of experiments: either transplanted, as small pieces, in male immunodeficient mice to generate xenografts or used freshly isolated single cells for in vitro and in vivo studies (Fig. 1).

For xenograft studies, we implanted tumor pieces (∼2–3 mm^3^) at three different anatomical sites [14], i.e., subcutis (s.c), kidney capsule (KC), or anterior prostate (AP) in one of the three immunodeficient mouse strains, i.e., NOD/SCID, Rag2^−/−^, or NOD/SCID-IL2Rγ^−/−^ (NSG) mice. NOD/SCID mice lack both T and B cells and have functional deficit (though not complete deficiency) in NK cells and Rag2^−/−^ mice also lack T and B cells whereas the NSG mice are the most immunodeficient lacking T, B, and NK cells [14], [47]. As summarized in Table 1 and detailed in Table S3, in every strain of mice and with every grade of HPCa, the s.c implants showed the highest tumor take compared to KC and AP xenotransplants. Furthermore, in NOD/SCID mice, which were used most often, we observed increasing tumor takes with increasing tumor grade at s.c and KC sites (Table 1; Table S3). Interestingly, the tumor grade-associated increase in tumor take was not observed in Rag2 and NSG mice, and HPCa samples xenotransplanted in NSG mice did not exhibit an increase in tumor takes compared to those implanted in NOD/SCID mice (Table 1; Table S3).

**Table 1:** HPCa xenotransplantation using tumor pieces in immunodeficient mice^a^.

HPCa(Gleason)	Number	Harvest time^b^(days)	Host	Tumor take (%)		
				s.c	KC	AP
GS6	6	175	N/S	8/16 (50)^#$^	2/26 (7.7)¶ ^$^	2/11 (18.2)@$
GS7	29	151	N/S	68/115 (59.1)^#&^	20/59 (33.9)¶ ^&^	1/20 (5)@&
	4	114	Rag2	14/22 (63.6)	–	–
	9	252	NSG	31/42 (73.8)	–	–
GS8	6	148	N/S	36/40 (90)# *	6/15 (40)¶ *	3/14 (21.4)@ *
	2	151	Rag2	5/8 (62.5)	2/2 (100)	–
	2	248	NSG	7/9 (77.8)	–	–
GS9	12	142	N/S	45/57 (78.9)^#^ §	6/12 (50)¶ §	4/10 (40)@ §
	1	258	Rag2	1/2 (50)	–	–
	7	237	NSG	29/38 (76.3)	–	–

a This table is summarized from Table S3. HPCa pieces (∼2–3 mm^3^) were implanted in the indicated strains of male immunodeficient mice supplemented with testosterone pellets. For s.c experiments, tumor pieces soaked in 50% Matrigel were surgically implanted. For KC experiments, tumor pieces were directly implanted in the kidney capsule of the host. For AP implantation experiments, tumor pieces were surgically grafted in the AP tubules. “−“, not done.

b Average time in days from the start of tumor piece implantation to the day of harvest.

c Tumor take refers to the number of tumors observed/number of implants.

# *P* = 0.0003;

¶ *P* = 0.023;

@ *P* = 0.13;

$ *P* = 0.006;

& *P* = 3.719e-06;

* *P* = 1.464e-06;

§ *P* = 0.01 (all conducted by Proportion test).

### HPCa Cells, Unlike HPCa Pieces, Failed to Re-initiate Tumors in NOD/SCID Mice: No Significant Effects with rUGM, CAFs, and Immortalized Human (Bone Marrow) Stromal (Hs5) Cells

Subsequently, we asked whether freshly isolated HPCa cells, rather than tumor pieces, could also regenerate tumors in any of the immunodeficient mice. In the very beginning, we recombined HPCa cells with rUGM for KC transplantations [48]–[50] or mixed the HPCa cells in 50% Matrigel for s.c injections in male NOD/SCID mice [14]. In 3 GS6 HPCa (HPCa9, 10, and 16) samples, when 10,000 to 100,000 HPCa cells recombined with rUGM were implanted under the KC, we observed 0/30 outgrowth (see Table 4 in ref. 14). Similarly, in 2 GS7 HPCa (HPCa11 and 14) samples, when 10,000 to 200,000 HPCa cells recombined with rUGM were implanted under the KC, we observed 0/17 outgrowth (Table 4 in ref. 14). Finally, when 1 million of HPCa18 (GS7) cells were injected s.c in 50% Matrigel, there was no single tumor out of 4 injections (Table 4; ref. 14). We then purified CD44^+^, CD133^+^ or CD44^+^CD133^+^ (and corresponding marker-negative) HPCa cells from 4 GS6 tumors (HPCa 9, 10, 13, and 16), 4 GS7 tumors (HPCa4, 6, 8, and 12), and 1 GS9 tumor (HPCa42) and implanted increasing numbers of cells (from 1,000 to 2 million) subcutaneously (for the GS9 HPCa cells) or in the KC or AP (for the rest) of the male NOD/SCID mice and we observed 0/225 outgrowths (Table 6 in ref. 14). We also purified CD44^+^/CD44^−^ HPCa cells from 3 GS8 (HPCa25, 32, and 33) and 1 GS9 (HPCa24) primary (1°) xenografts and implanted 1,000 to 500,000 cells in 50% Matrigel at the most sensitive site, i.e., subcutis of male NOD/SCID mice. Again, we did not observe any tumor regeneration out of 52 implantations (Table 6 in ref. 14). Finally, when unsorted HPCa cells purified from 3 GS6 tumors (HPCa2, 10, and 16), 4 GS7 tumors (HPCa3, 11, 14, and 18), 2 GS8 tumors (HPCa15 and 37), and 2 GS9 tumors (HPCa5 and 21), either injected subcutaneously alone in Matrigel or recombined with rUGM and then transplanted under the KC, gave rise to 0/92 tumors (Table 7 in ref. 14). In all these transplantation experiments, the male NOD/SCID mice were supplemented with exogenous testosterone pellets.

Next, we co-injected unsorted or marker-sorted HPCa cells with CAFs, which have been reported to promote tumor development in some experimental systems [51], either subcutaneously or under the KC of testosterone-supplemented male NOD/SCID mice. We observed 3 outgrowths out of a total of 56 injections (Table S4). Nevertheless, the 3 outgrowths were not serially transplantable (Table S4).

Recently, human bone marrow derived mesenchymal stromal (or stem) cells (MSCs) have been shown to integrate into the tumor-associated stroma and promote breast cancer cell metastasis [52]. We wondered whether MSCs might facilitate tumor reconstitution by primary HPCa cells. The Hs5 cell line, generated from human bone marrow, was immortalized by transduction with human papilloma virus E6/7 genes [44], and has been shown to support proliferation of PCa cells in cultures [53], [54]. We thus subcutaneously coinjected unsorted or marker-sorted (i.e., CD44) HPCa cells (from 2 GS6, 3 GS7, 4 GS8, and 1 GS9 tumors) with 100,000 Hs5 cells into NOD/SCID mice. We observed 4 tumors in a total of 54 injections (Table S5). Again, the 4 regenerated tumors could not be serially transplanted (not shown). Hs5 cells alone did not generate tumors in NOD/SCID mice at ≤ 100,000 cells (data not shown).

Taken together, our prior (ref. 14, Tables 4, 6, and 7) and current (Table S4 and S5) studies indicate that *NOD/SCID mice are not permissive for reconstituting transplantable tumors from primary HPCa cells, even in the presence of rUGM, CAFs, or MSCs such as Hs5*.

### Hs5 Cells Induce HPCa Cells to Initiate Transplantable Tumors in NSG Mice

What if we utilize more immunodeficient NSG mice? We first injected freshly purified primary HPCa cells from 5 patient tumors (2 GS7, 1 GS8, 2 GS9) subcutaneously into NSG mice. In a total of 37 injections, we did not observe any tumor growth after 8 months (Table 2). Since Hs5 cells slightly increased tumor regeneration of HPCa cells in NOD/SCID mice (Table S5), we subcutaneously coinjected unsorted HPCa cells from 9 patient samples with Hs5 cells into NSG mice and, remarkably, this modified ‘recombination protocol’ induced tumor formation in 41/59 (i.e., ∼70%) injections (Table 3). HPCa cells derived from 3 GS9, 1 GS8, and 4 GS7 tumors all regenerated tumors when coinjected with Hs5 cells (Table 3). We also established 1° xenografts in Rag2 mice from pieces of 3 other HPCa samples (i.e., HPCa57, 58, and 70). When the 1° xenograft cells were purified out and coinjected with Hs5 cells into male Rag2 or NSG mice, we readily obtained the 2° xenografts [21, 25; data not shown]. In total, we have established 11 HPCa/Hs5 xenografts from 7 GS7 (HPCa57, 58, 70, 83, 84, 85, and 92), 1 GS8 (HPCa91), and 3 GS9 (HPCa80, 87, and 96) tumors. These results seem to suggest that Hs5 cells highly efficiently promote tumor regeneration in NSG mice from fresh HPCa cells.

**Table 2:** Freshly purified and unsorted HPCa cells injected in NSG mice^a^.

Sample	Cell number (# injections)	Harvest(days)	Incidence
HPCa84 (GS7)	12.5k (1x), 50k (1x), 100k (1x)	177	0/3
HPCa85 (GS7)	100k (1x), 200k (1x), 500k (1x)	160	0/3
HPCa91 (GS8)	100 (3x),1k (3x), 10k (3x), 50k (1x)	123	0/10
HPCa87 (GS9)	1k (4x), 10k (5x), 100k (3x), 300k (1x)	143	0/13
HPCa89 (GS9)	1k (2x), 10k (2x), 100k (2x), 1M (2x)	248	0/8
		** Total: 0/37**	

a Freshly purified and unsorted HPCa cells were subcutaneously injected in 50% Matrigel in 6–8 week old male NSG mice supplemented with testosterone pellet.

**Table 3:** HPCa cells mixed with Hs5 cells initiate serially transplantable tumors in NSG mice*.

HPCa sample	Cell number	Harvest time (d)	Incidence
HPCa82 (GS6)	10k (2x), 100k (1x)	272	0/3
HPCa83 (GS7)	10k (5x)	185	5/5
HPCa84 (GS7)	12.5k (1x), 50k (1x), 100k (1x)	185	2/3
HPCa85 (GS7)	100k (1x), 200k (1x), 500k (1x)	160	3/3
HPCa92 (GS7)	1k (2x), 10k (2x), 100k (2x), 500k (2x)	149	5/8
HPCa91 (GS8)	100 (3x), 1k (3x), 10k (3x), 50k (1x)	154	9/10
HPCa80 (GS9)	1k(2x), 10k(2x), 30k(1x), 300k(1x)	147	4/6
HPCa87 (GS9)	1k (4x), 10k (5x), 100k (3x), 300k (1x)	143	7/13
HPCa96 (GS9)	1k (2x), 10k (2x), 100k (2x), 500k (2x)	161	6/8
		** Total: 41/59 = 69.5%**	

* Freshly purified and unsorted HPCa cells at the indicated numbers were recombined with 100,000 Hs5 cells and then subcutaneously injected in 50% Matrigel in 6–8 week old male NSG mice supplemented with testosterone pellet.

The *reconstituted* tumors were highly tumorigenic and could be passaged indefinitely (Table S6; data not shown). Also, the regenerated tumors were able to give rise to transplantable tumors independently of Hs5 cells (Table S6). Moreover, unsorted or CD44-sorted (both CD44^+^ and CD44^−^) HPCa cells were able to re-initiate tumors both subcutaneously and in the dorsal prostate (DP) and, significantly, HPCa cells purified from the xenografts initially established in NSG mice could regenerate tumors in NOD/SCID mice (Table S6; data not shown).

### Reconstituted “Prostate” Tumors are of the Human Origin and Present an Undifferentiated and Epithelial Morphology: IHC, Biochemical, and Molecular Characterizations

**Figure 2:**
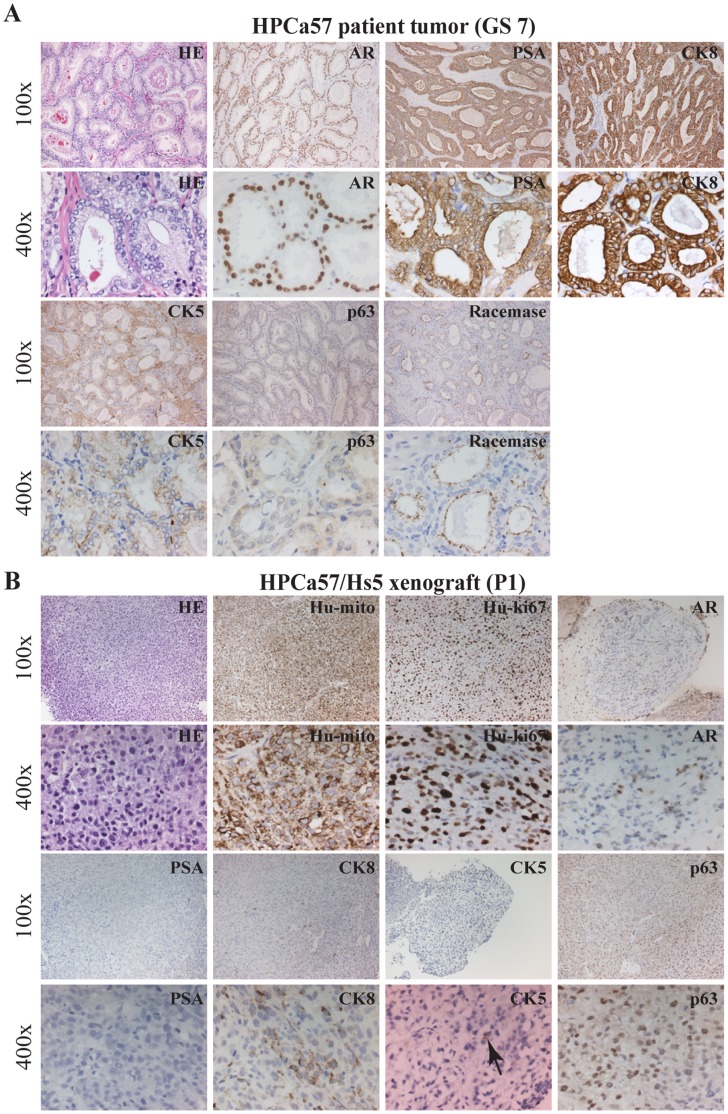
IHC analysis of HPCa57 patient sample (GS7) and its xenograft tumor. (A) Staining of HE, AR, PSA, CK8, CK5, p63 and Racamase in HPCa57 patient sample. (B) HPCa57P1 xenograft tumor, derived from coinjection with 100,000 Hs5 cells, was used to make serial sections, which were stained for HE, Hu-mito, Hu-ki67, AR, PSA, CK8, CK5 and p63. Both low (i.e., 100x) and high-power (i.e., 400x) magnifications were shown. Arrow indicates CK5^+^ cells.

**Figure 3:**
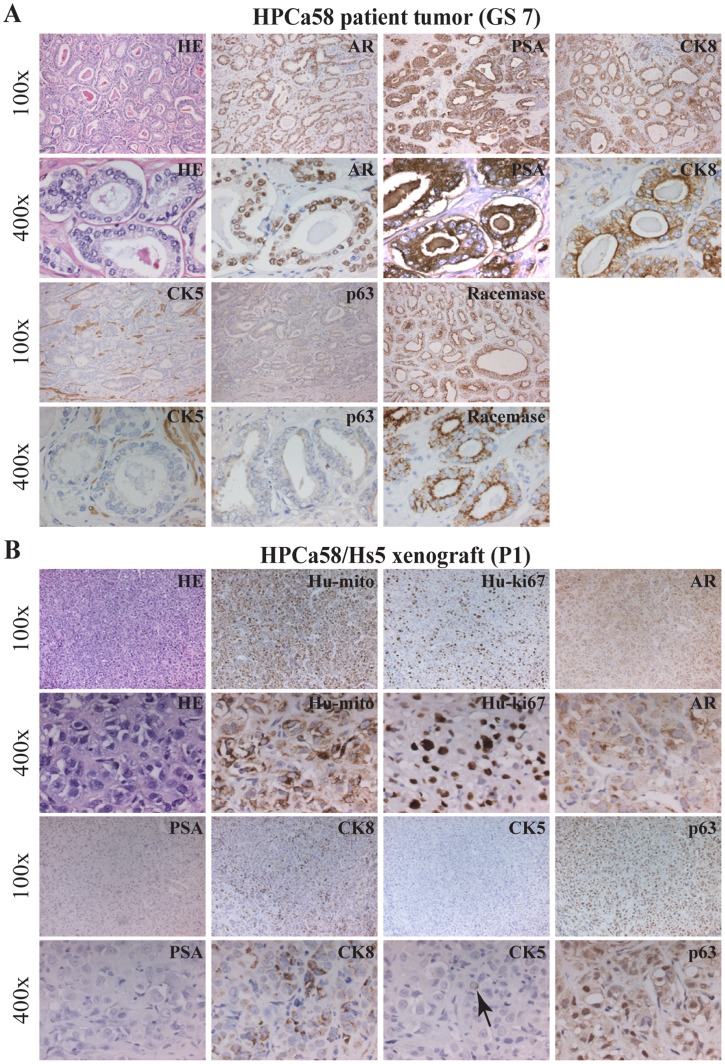
IHC analysis of HPCa58 patient sample (GS7) and its xenograft tumor. (A) Staining of HE, AR, PSA, CK8, CK5, p63 and Racamase in HPCa58 patient sample. (B) HPCa58P1 xenograft tumor, derived from coinjection with 100,000 Hs5 cells, was used to make serial sections were stained for HE, Hu-mito, Hu-ki67, AR, PSA, CK8, CK5 and p63. Both low (i.e., 100x) and high-power (i.e., 400x) magnifications were shown. Arrow indicates CK5^+^ cells.

We first carried out histological and IHC characterizations of the reconstituted tumors. As expected, the HPCa57 patient tumor exhibited typical GS7 histology with crowded tumor glands, in which most cells stained positive for prostate luminal epithelial cell markers PSA, nuclear AR, and cytokeratin 8 (CK8) (Fig. 2A). Also, tumor glands showed positive staining for racemase (alpha-methylacyl-CoA racemase, also known as AMACR or P504S; encoded by the *AMACR* gene), negative (or weakly positive) staining for CK5 (a prostate basal epithelial marker), and negative staining for p63 (another basal cell marker) (Fig. 2A). The HPCa57/Hs5 reconstituted tumors in NSG mice appeared completely undifferentiated and lacked glandular structures and PSA expression (Fig. 2B). The cells looked overall epithelial, as supported by the presence of some CK8^+^ and sporadic CK5^+^ cells (Fig. 2B). Interestingly, scattered AR^+^ and p63^+^ cells could be observed (Fig. 2B). Staining with human-specific antibodies against mitochondria and Ki-67, both of which did not stain mouse tissues (Fig. S1), confirmed the human origin of the regenerated HPCa57 tumor (Fig. 2B). Similar patterns of morphology and marker expression were observed in Hs5-reconstituted HPCa58 (Fig. 3) and 9 other HPCa samples, i.e., HPCa70, 80, 83, 84, 85, 87, 91, 92, 96 (data not shown).

**Figure 4:**
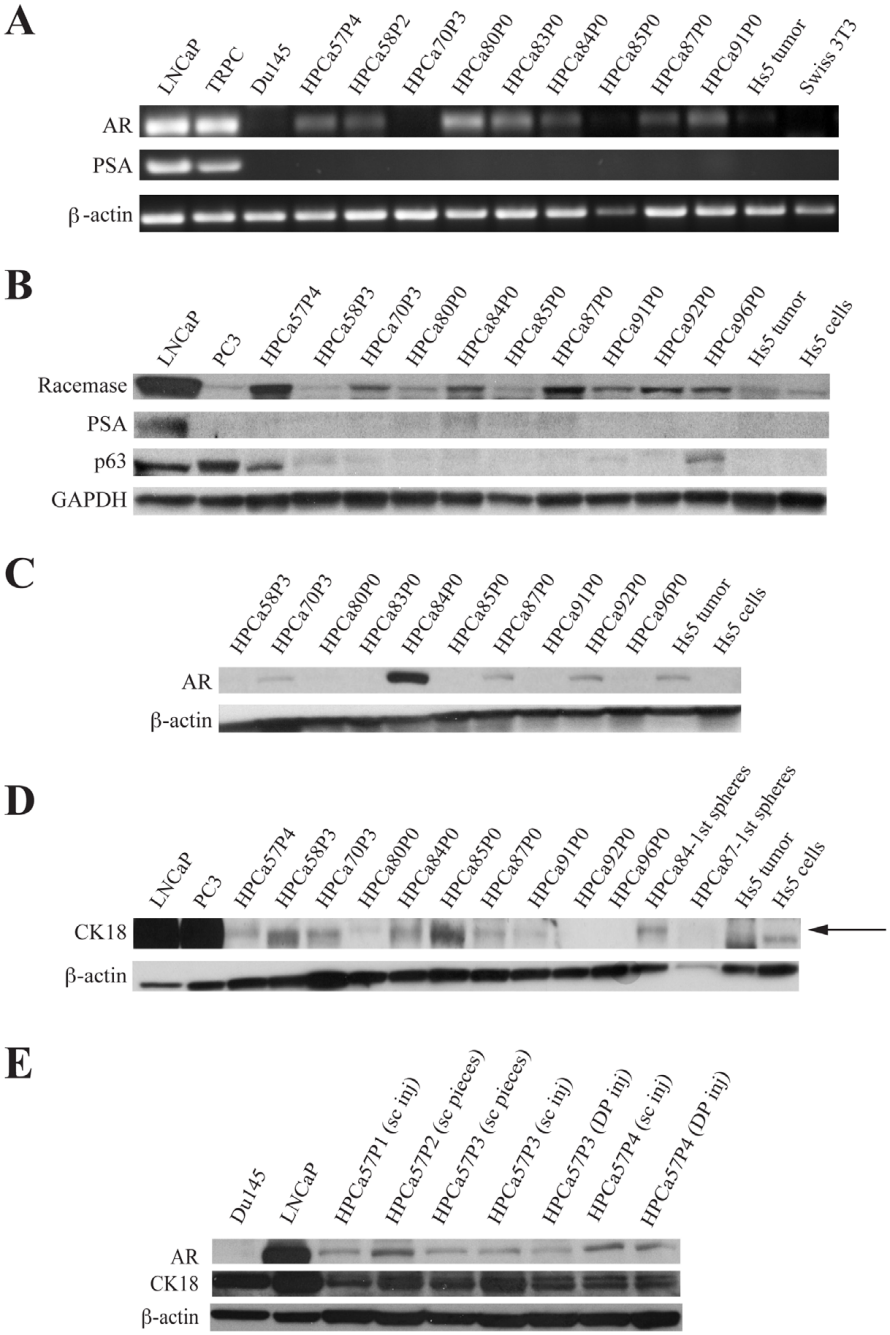
RT-PCR (A) and Western blotting (B–E) characterizations of HPCa/Hs5 xenograft tumors. (A) RT-PCR results of AR, PSA, and β-actin using human-specific primers. LNCaP, TRPC (treatment-refractory prostate cancer; 46) and Du145 cells were used as controls. (B–D) Western blotting of Racemase, PSA, p63, CK18 and AR on HPCa-Hs5 xenograft tumors and the tumor cell-derived spheres. GAPDH and β-actin were used as loading controls. The arrow in D indicated the CK18 protein band (note that Hs5 cells and Hs5 tumor had some non-specific lower M.W bands). (E) Western blotting of AR, CK18 and β-actin in HPCa57/Hs5 serial xenograft tumors (P1–P4) from either subcutaneous or orthotopical (DP) injections. LNCaP and Du145 cells were used as controls.

Consistent with the IHC results, RT-PCR analysis using human-specific primers revealed that none of the HPCa/Hs5 xenografts expressed *PSA* but most expressed different levels of human *AR* mRNA (Fig. 4A). Notably, cultured murine fibroblasts (Swiss 3T3) and Hs5 cells were negative for both *AR* and *PSA* mRNAs (Fig. 4A). Western blotting experiments for 4 luminal cell markers, racemase, PSA, AR, and CK18 confirmed lack of PSA expression and revealed varying levels of the other 3 markers in different xenografts (Fig. 4B–D). Note that cultured Hs5 cells and Hs5 tumors (see below) did not express CK18 (Fig. 4D, arrow) although they expressed low levels of racemase (Fig. 4B). Occasionally, Hs5 tumors were found to express low levels of AR protein (Fig. 4C) although they expressed barely detectable *AR* mRNA (Fig. 4A). Most HPCa/Hs5 xenografts expressed higher levels of racemase than PC3 cells (Fig. 4B). Interestingly, Western blotting for p63 revealed very high levels in PC3 and LNCaP cells, readily detectable expression in HPCa57 and HPCa96 xenografts, and very low levels in several other HPCa xenografts (HPCa58, 70, and 91) (Fig. 4B). These results overall suggest that the *HPCa/Hs5 xenografts contain human prostatic epithelial cells*. As further support, Western blotting of AR and CK18 on a series of HPCa57/Hs5 xenograft tumors, from the 1° generation (P1) to the 4° generation (P4), derived from s.c or DP implantations, showed that all these tumors were positive for AR and CK18 (Figure 4E).

### Cytogenetic Evidence for Human PCa Cells in HPCa/Hs5 Xenograft Tumors and Physical Contributions of Hs5 Cells to the Xenografts

**Figure 5:**
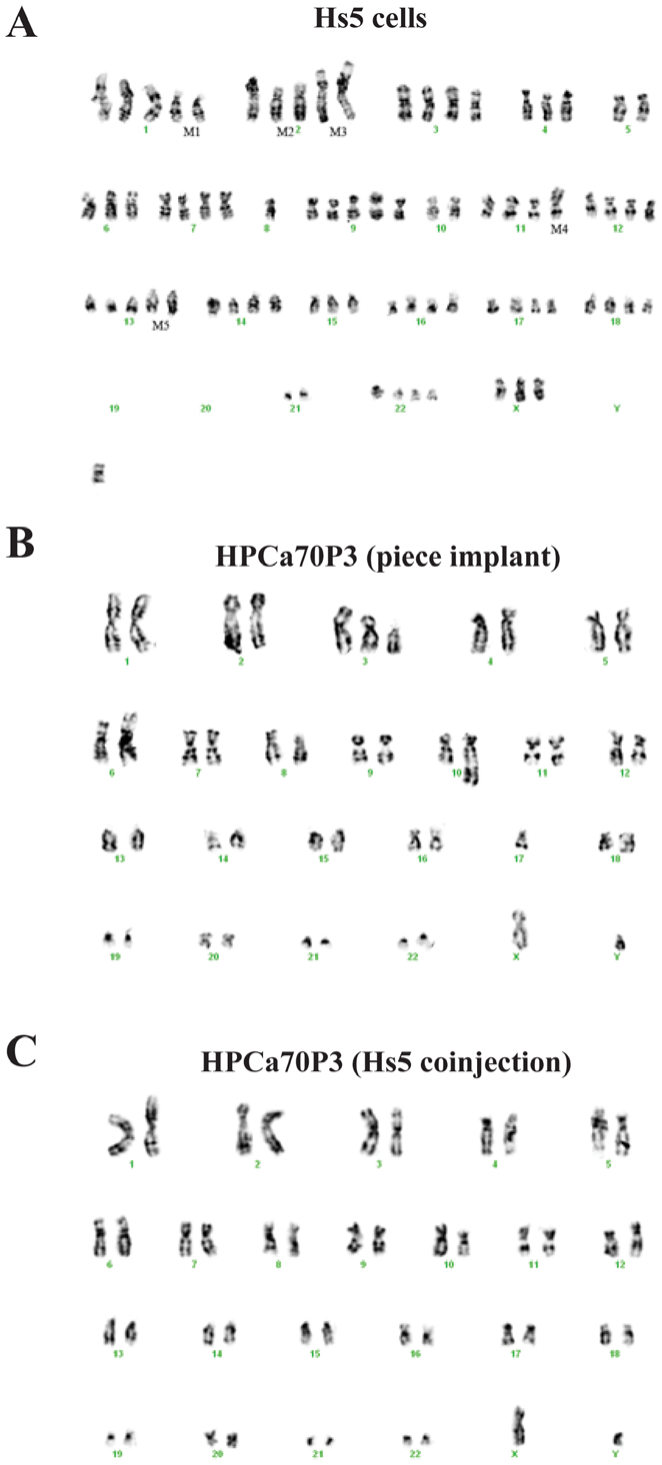
Cytogenetic analysis of HPCa/Hs5 xenograft tumors. (A) An example of Hs5 cell karyotype. (B–C) Karyotypes of xenograft cells derived from HPCa70 piece implantation (B) or from HPCa70/Hs5 coinjections (C).

Our cytogenetic analyses (Fig. 5) provided further evidence for the conclusion that HPCa/Hs5 xenografts contain human prostatic epithelial cells. The cultured Hs5 cells showed multiple chromosomal abnormalities with at least 5 marker chromosomes, i.e., M1 [del(1p)], M2 [del(2p)], M3 [der(2)], M4 [der(11)], and M5 [13q+] (Fig. 5A). HPCa70 cells derived from either tumor piece implant (Fig. 5B) or Hs5 coinjections (Fig. 5C) showed similar karyotypic features that were distinct from the cytogenetic makeup of Hs5 cells. Specifically, tumor cells from both HPCa70/Hs5 coinjections and tumor piece implants showed a deletion at chromosome 10, which is a common chromosomal abnormality in PCa [55]–[57].

On the other hand, when we performed similar cytogenetic analysis in cells derived from HPCa57/Hs5 and HPCa87/Hs5 xenografts, most cells we were able to karyotype showed karyotypic features similar to those of Hs5 cells (not shown), suggesting that epithelial human PCa cells in these tumors represented the minority with Hs5 cells being the majority. Also, the overall histological and structural dissimilarities between the HPCa/Hs5 xenografts and the corresponding patient tumors made us wonder whether Hs5 cells might have physically contributed to the establishment of the xenografts. In partial support of this conjecture, Hs5 cells injected alone were capable of initiating tumor development in NSG mice with a grafting efficiency at ∼ 52.5% (Table S7). Both Hu-mito and Hu-Ki67 staining of the Hs5 cell-derived tumors was positive (Fig. S2, top), confirming their human origin. The Hs5 tumors manifested a stromal morphology, and IHC staining of these tumors was completely negative for PSA, CK8, CK5 and p63 (Fig. S2), consistent with RT-PCR and Western blotting results (Fig. 4A, B, and D). AR staining was observed in some cells but only in the cytoplasm (Fig. S2). These data, taken together, suggest that Hs5 cells were tumorigenic in the highly immunodeficient NSG mice, but tumors derived from these cells were distinct from HPCa/Hs5 xenograft tumors.

### The Reconstituted HPCa/Hs5 Tumors Contain EpCAM^+^ Epithelial Cancer Cells that can Regenerate Tumors that Contain both Epithelial and Mesenchymal-like Cells

**Figure 6:**
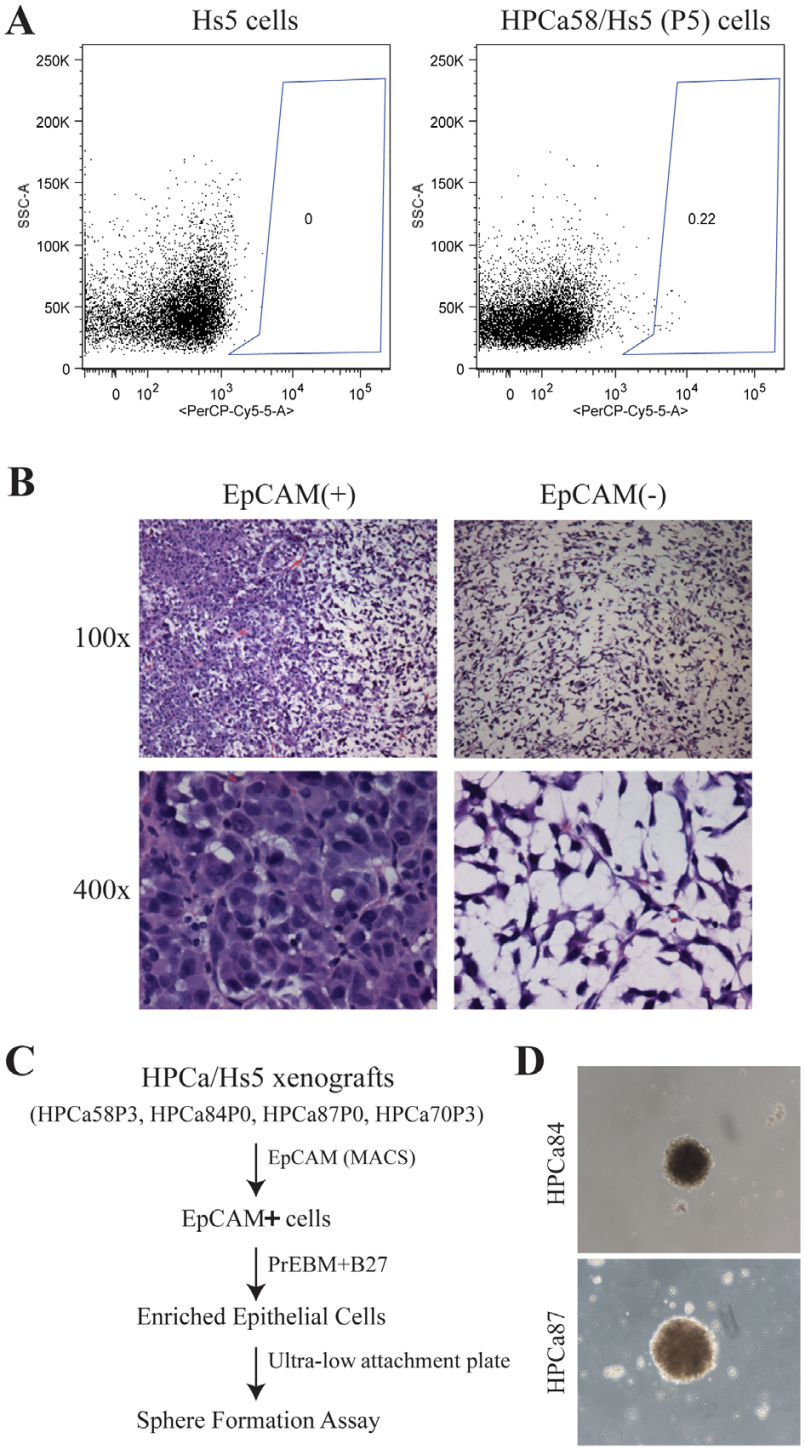
Functional characterizations of EpCAM^+^ cells in HPCa/Hs5 tumors. (A) FACS analysis of EpCAM expression in cultured Hs5 cells and tumor cells harvested from HPCa58P/Hs5 tumors (P5). (B) HE staining of tumors derived from 100 EpCAM^+^ and EpCAM^−^ cells from HPCa58/Hs5 tumors. (C) Scheme of sphere formation assays using HPCa/Hs5 xenograft tumor cells. (D) Representative images of spheres derived from EpCAM^+^ HPCa84 and HPCa87 tumor cells (100x).

To provide further evidence that reconstituted HPCa/Hs5 tumors contain epithelial cells, we analyzed the expression of epithelial cell adhesion molecule (i.e., EpCAM) and detected a small percentage of EpCAM^+^ cells in the HPCa/Hs5 tumors (Figure 6A; data not shown). To functionally analyze these EpCAM^+^ cells, we purified out EpCAM^+^ and isogenic EpCAM^−^ cells from HPCa58/Hs5 tumors and injected them into male NSG mice. Interestingly, the EpCAM^+^ cells in HPCa58/Hs5 tumors were capable of regenerating tumors with as few as 100 cells, and, importantly, the EpCAM^+^ cell-derived tumors comprised both epithelial- and stromal-like cells (Fig. 6B). In contrast, EpCAM^−^ cells gave rise to tumors consisting of only stromal-like cells (Fig. 6B).

We also sorted out EpCAM^+^ cells from HPCa58/Hs5 tumors via Magnetic Activated Cell Sorting (MACS), cultured them in PrEBM medium in collagen treated plates, and, finally, tested their sphere-forming capacities (Fig. 6C). We observed that most EpCAM^+^ cells attached and proliferated to give rise to cells that were capable of generating serially passageable spheres in the ultra-low attachment plates (Fig. 6D). Importantly, some of these spheres were positive for CK18 (Fig. 4D). Taken together, these results further indicate that *the reconstituted HPCa/Hs5 tumors contain a subset of epithelial (EpCAM^+^) PCa cells that are clonogenic as well as tumorigenic*.

### Evidence that Undifferentiated HPCa Cells Might be the Cells that Reconstituted the HPCa/Hs5 Tumors

**Figure 7:**
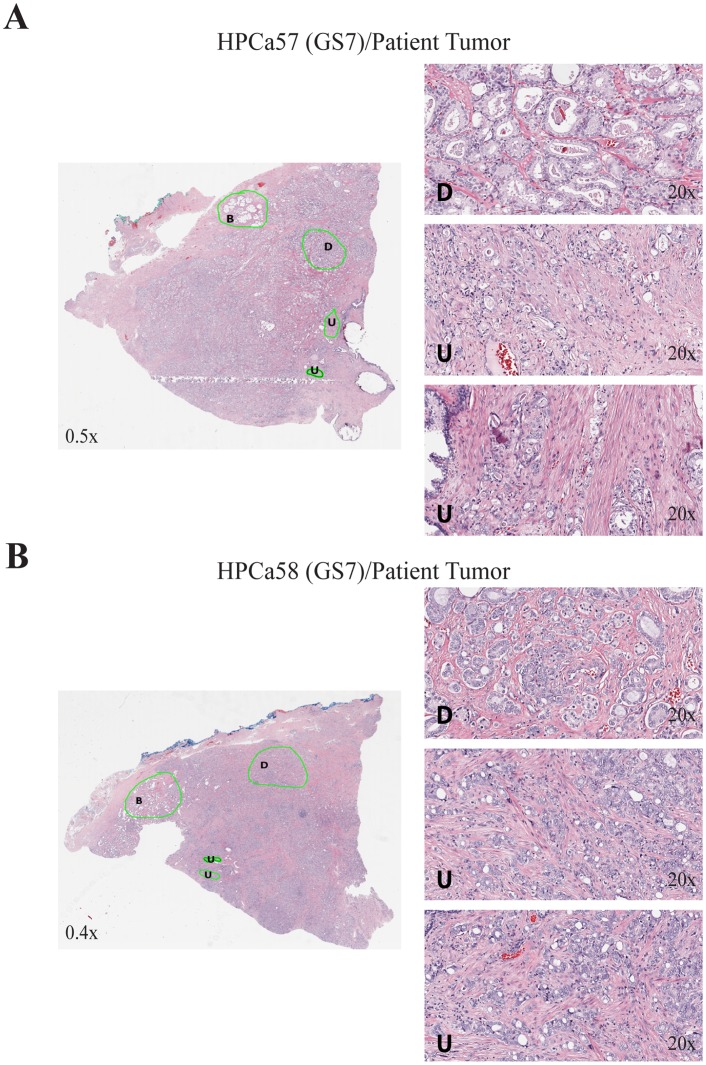
Histological and cellular heterogeneity in GS7 prostate tumors. Shown are whole-mount Aperio ScanScope images of HPCa57 (A) and HPCa58 (B) patient tumors, in which benign (B) glands, differentiated (D), and undifferentiated (U) tumor areas can be identified. Enlarged images of one differentiated and two undifferentiated areas are shown on the right. The magnifications of the original objectives are indicated.

**Figure 8:**
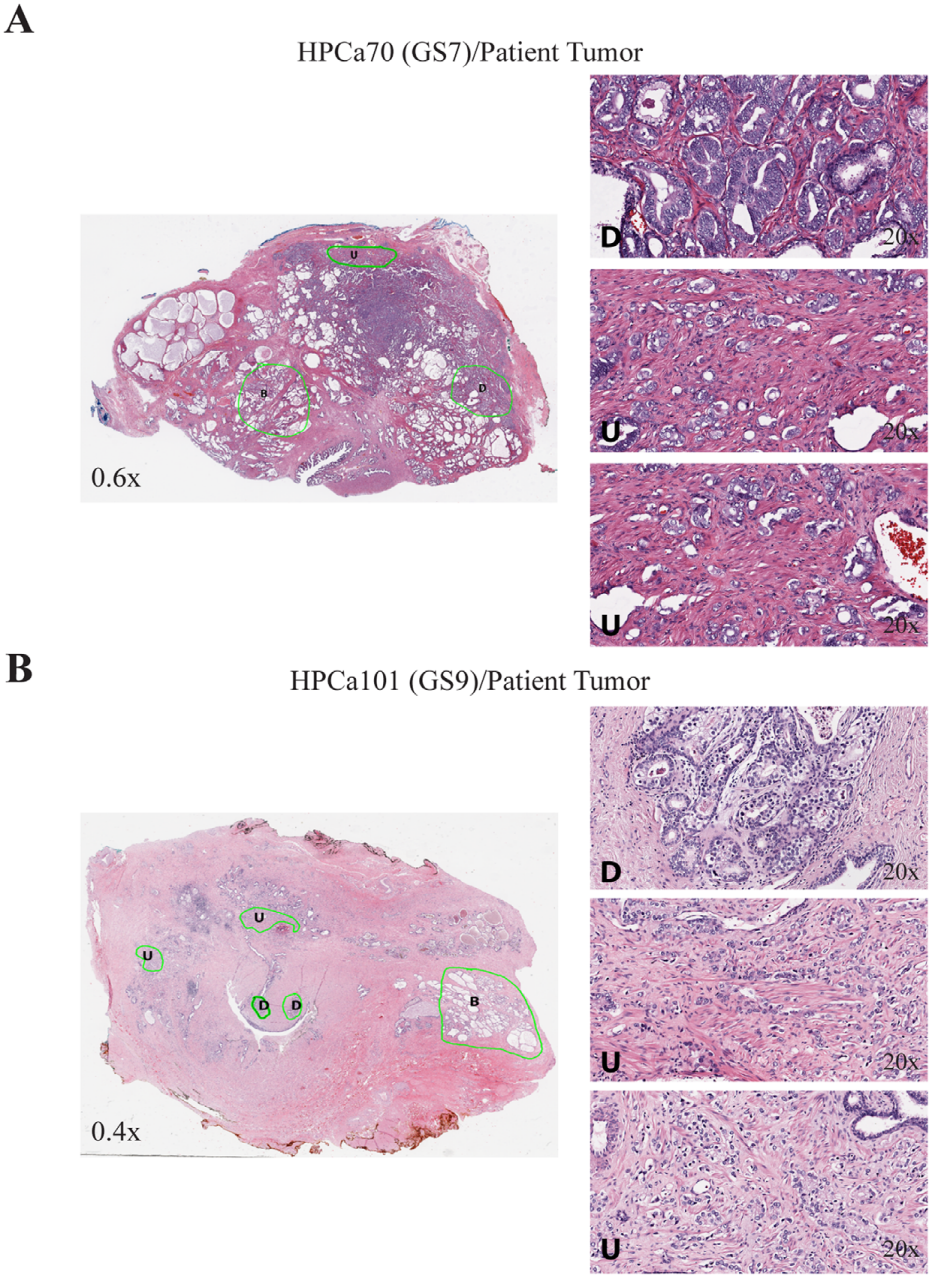
Histological and cellular heterogeneity in GS7 and GS9 prostate tumors. Shown are whole-mount Aperio ScanScope images of HPCa70 (A) and HPCa101 (B) patient tumors, in which benign (B) glands, differentiated (D), and undifferentiated (U) tumor areas can be identified. Enlarged images of one differentiated and two undifferentiated areas are shown on the right. The magnifications of the original objectives are indicated.

The observations that GS7 tumors such as HPCa57 and HPCa58, which contained well-differentiated glandular structures, nevertheless reconstituted tumors as fully undifferentiated tumors (Fig. 2; Fig. 3) are intriguing. When the whole-mount sections were analyzed under Aperio ScanScope, we observed discernible regions of poorly differentiated or undifferentiated tumor cells in all GS7 tumors such as HPCa57 (Fig. 7A), HPCa58 (Fig. 7B), and HPCa70 (Fig. 8A). On the other hand, although most tumor cells in GS9/10 tumors were poorly differentiated or undifferentiated, differentiated areas with glandular structures could clearly be observed (e.g., HPCa101; Fig. 8B). These cellular and structural heterogeneities in patient prostate tumors raised a possibility that undifferentiated HPCa cells might be the cells that survived in the tumor microenvironment and gave rise to transplantable xenograft tumors in the presence of Hs5 cells.

**Figure 9:**
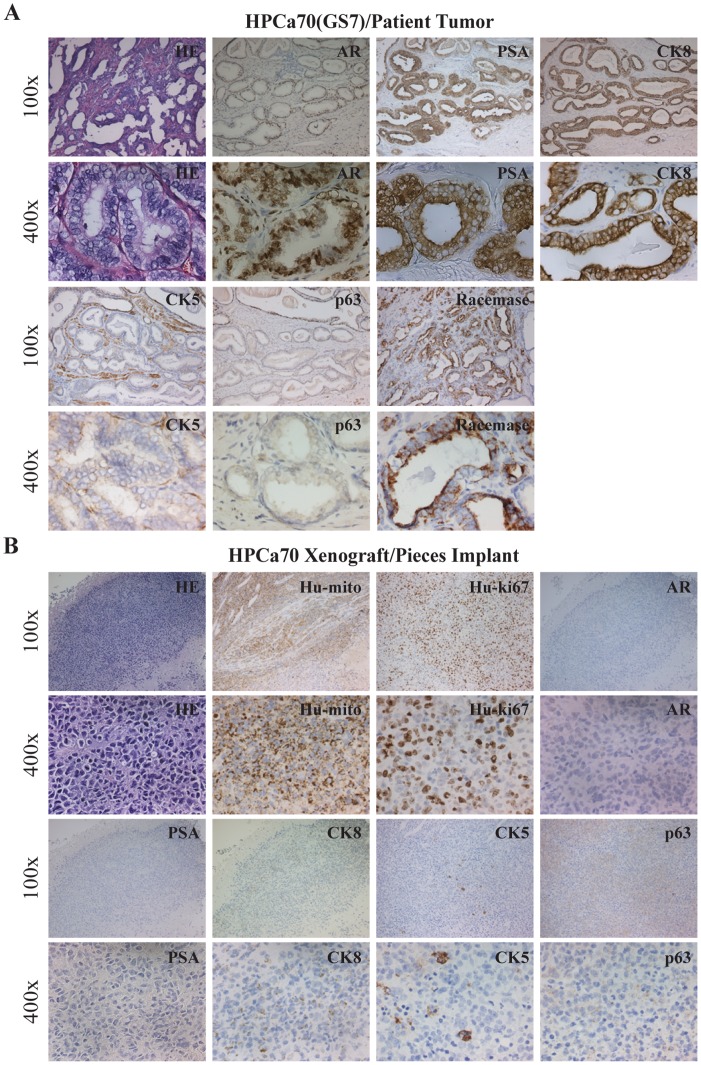
Histological analysis of HPCa70 patient sample (A) and its piece implant-derived xenograft tumor (B). Molecules stained and original magnifications are indicated.

**Figure 10:**
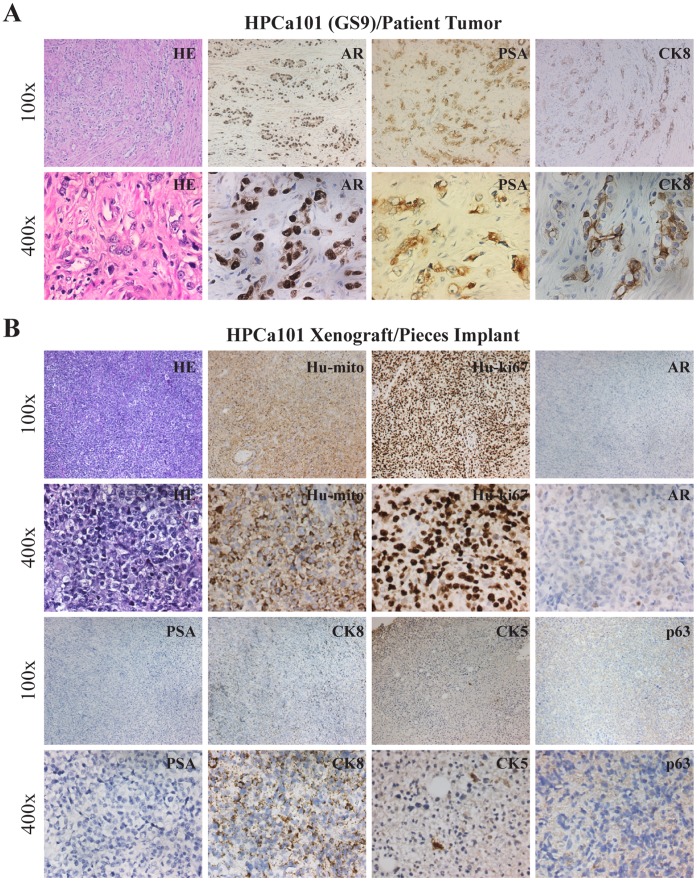
Histological analysis of HPCa101 patient sample (A) and its piece implant-derived xenograft tumor (B). Molecules stained and original magnifications are indicated.

As indirect support for this possibility, we have successfully established two “pure” HPCa xenograft lines, i.e., HPCa70 and HPCa101 that were derived from primary tumor pieces implanted subcutaneously into the NSG mice. In HPCa70 patient tumor, most tumor cells were highly positive for luminal markers AR, PSA, CK8, and racemase but weakly positive for CK5 and negative for p63 (Fig. 9A). Strikingly, the HPCa70 xenograft tumor, which was established by implanting tumor pieces without Hs5 cells presented a fully undifferentiated morphology and IHC staining was negative for PSA and weakly positive for AR (Fig. 9B). The tumor piece-derived xenograft tumors were expectedly of the human origin (Hu-ki67^+^, Hu-mito^+^) and contained CK8^+^ and CK5^+^ cells (Fig. 9B). Similarly, in the HPCa101 patient tumor (GS9), most tumor cells were positive for nuclear AR but moderately positive for CK8 and PSA, perhaps due to their overall poorly differentiated nature (Fig. 10A). The corresponding HPCa101 xenograft tumor derived from pieces implant showed an undifferentiated morphology, was negative for PSA and p63 and weakly positive for AR, and contained CK8^+^ and CK5^+^ epithelial cells (Fig. 10B). These interesting observations in two tumor pieces-derived xenograft tumors, which histologically and immunophenotypically resembled the HPCa/Hs5 reconstituted xenograft tumors, raise the possibility that undifferentiated HPCa cells might have a survival advantage to regenerate tumors.

## Discussion

The PCa field has long been hampered by the paucity of suitable xenograft models. There are two lines of studies that attempt to reconstitute human PCa development in immunodeficient mice, i.e., tumor piece implantation and cell injection. In the past, PCa xenografts from either way were mainly derived from metastasis [36]–[38]. It is conceivable that more challenges exist to establish xenografts using single cells derived from primary human PCa samples in that many factors will contribute to the complexity of reconstitution such as tumor grade, tumor type, relative abundance of tumor cells, host, applied methodologies, etc [14]. As a result, tumor piece implantation has been used more widely. It is believed that such “tumorgrafts” should recapitulate primary patient tumors histopathologically and molecularly [58], at least to a certain degree.

Various groups have attempted to establish HPCa xenograft models by using tumor piece implantation. For example, Wang et al compared the efficiency and histopathologic patterns of xenografting both benign and malignant human prostate tissue (low- to mid-grade) into different sites (subrenal, orthotopic and subcutaneous) of SCID mice, and they showed that both subrenal capsule and orthotopic sites could be used for HPCa xenograft studies with respect to high take rate and histopathologic differentiation [59]. More recently, Priolo et al. implanted 30 primary localized prostate tumor pieces into the KC site of Nu/Nu or NOD/SCID mice, and they obtained a 56% tumor take with very low tumor take from subcutaneous and orthotopic implantations [41]. The xenografts from subrenal site maintained both grading and expression of phenotypic markers of the parental patient tumors [41]. Also, a tissue slice graft model has been developed by subrenal implantation of fresh thin, precision-cut tissue slices derived from 2 primary patient adenocarcinomas into RAG2-γ mice, and this model has been advocated as a tool to model all stages of PCa [42]. Most of these implantations have been done in the subrenal site, in which tumor growth is somewhat limited. Also, whether these grafted tumors can be serially transplanted remains unknown. In our study, we implanted primary tumor pieces from 78 untreated patients (ranging from GS6-GS9) into the male NOD/SCID, Rag2, or NSG mice supplemented with testosterone at 3 different sites (i.e., SC, KC and/or AP) (Table 1 and Table S3). Our results reveal the subcutaneous site to be the most sensitive in NOD/SCID mice in allowing tumor piece grafting. In addition, tumor grade positively correlates with tumor take at the subcutaneous site. Importantly, we have established two serially transplantable xenograft models from two primary patient samples using subcutaneous tumor piece implantation, i.e., HPCa70 (GS7) and HPCa101 (GS9) (Fig. 9 and 10). To our knowledge, these two xenograft lines are among the few serially transplantable PCa models that originate from primary patient tumors.

Reconstitution of PCa in the immunodeficient mice from patient-derived HPCa single cells is much more challenging, explaining, partially, why there is a limited number of PCa cell lines currently available [60]. This challenge is also the underlying reason why it has yet to be demonstrated that primary human PCa cells contain stem-like cancer cells that can initiate serially transplantable xenograft tumors, a gold standard to functionally characterize CSCs in vivo [61], although such studies have been done with many PCa xenograft models or cultured cell lines (see Introduction). It is rather striking how indolent primary PCa cells are compared to many other tumor cells such as melanoma, colorectal cancer, and glioblastoma cells, which, when freshly purified from patient tumors and implanted in Matrigel in immunodeficient mice, can readily regenerate xenograft tumors that even frequently resemble the donor patient tumor histologies [33], [47]. In sharp contrast, acutely purified HPCa cells, either bulk or marker-enriched, are virtually non-tumorigenic when implanted in Matrigel in NOD/SCID or NSG mice, even in the presence of “helpers” such as rUGM and CAFs [14; this study].

There are several novel and important findings from our current study. *First*, bulk or marker-sorted HPCa cells, when injected alone in 50% Matrigel, cannot induce tumor growth, even in the highly immunodeficient NSG mice, supporting the notion that HPCa cells are quite indolent. These results also suggest that either primary HPCa cells need special microenvironment to maintain their growth in vivo, or they need manipulations to enhance their tumor-initiating potential. About this latter point, several groups have taken the lead [62]–[64]. For example, Goldstein et al recently showed that basal cells from primary benign human prostate tissues are capable of initiating PCa in NSG mice upon overexpressing 3 oncogenic molecules (i.e., AKT, ERG, and AR) [63], [64].

*Second,* although HPCa cells coinjected with rUGM, CAFs, or Hs5 cells do not regenerate serially transplantable tumors in NOD/SCID mice after 6–9 months, Hs5 cells significantly enhance the ability of HPCa cells to initiate serially transplantable tumors in NSG mice (∼10-fold increase compared to in NOD/SCID mice). Thus, our results in PCa confirm that more immunodeficient mice dramatically increase primary tumor take/incidence, as shown in melanoma [47]. Furthermore, our study hints that certain microenvironments (e.g., coinjection of ‘helper’ cells) may likely help primary HPCa cells set a foothold in vivo. To our knowledge, our work is the first in the field to systematically compare tumor take/incidence in NOD/SCID versus NSG mice by using patient-derived HPCa single cells.

*Third,* recent evidence suggests that bone marrow-derived human MSCs increase tumor grafts of human breast cancer cells by promoting angiogenesis [65] as well as enhance metastatic capacities [52]. Here we report, for the first time, that the immortalized human MSCs (i.e., Hs5) reliably promote human prostate tumor reconstitution in NSG mice. Several pieces of evidence support the presence of epithelial PCa cells in the reconstituted HPCa/Hs5 tumors: most tumor cells present an epithelial morphology; CK8^+^ and CK5^+^ cells can be observed; RT-PCR and/or Western analyses reveal AR and CK18 expression in most tumors; karyotyping analysis shows cytogenetic abnormalities characteristic of human PCa cells; and presence of EpCAM^+^ cells that are both clonogenic and tumorigenic.

Intriguingly, all HPCa/Hs5 tumors, including those from GS7 tumors, present a fully undifferentiated histology lacking glandular structures. Consistent with the undifferentiated tumor histology, all HPCa/Hs5 tumors lack PSA and only express very low levels of AR. These results suggest that Hs5 cells fail to fully reconstitute the original patient tumor histology from disaggregated primary HPCa cells. The fact that prostate tumors are extremely heterogeneous (Fig. 7 and 8) leads us to propose that it is perhaps only the undifferentiated PCa cells in the primary tumors that have the ability to reconstitute tumor formation in highly immunodeficient mice. As indirect support for this proposal, undifferentiated (i.e., PSA^−/lo^), compared to differentiated (PSA^+^) PCa cells, are enriched in prostate CSCs that possess long-term tumor-propagating capacity [25]. As further support, the phenotype of “undifferentiation” in our HPCa/Hs5 reconstituted tumors is similar to that in xenograft tumors derived from HPCa70 and HPCa101 tumor pieces. In fact, it has been reported that subcutaneous transplantation of primary PCa pieces into nude mice leads to xenograft tumors composed entirely of undifferentiated cells [66].

The exact mechanisms by which Hs5 cells support HPCa tumor regeneration need further investigation. One possibility is that Hs5 cells secrete critical cytokines such as IL-6 that help maintain the survival of undifferentiated PCa cells. Another possibility is that Hs5 cells promote HPCa tumor reconstitution via cell-cell fusion as we have demonstrated that prostatic epithelial cells and fibroblasts have a high propensity to fuse with cancer cells [67]. Placencio et al also reported, recently, that MSCs from FSP1-Cre/Rosa 26 mice were recruited to the prostate and could fuse with local prostate epithelial cells from β-actin-GFP mice, manifested by co-expression of β-galactosidase and GFP during prostate regrowth after castration [68]. In addition, MSCs were able to home to C4-2B xenograft tumors and enhance Wnt signaling activity [68]. Using a co-culture model, Wang et al found that some of the cancer-stromal hybrids could survive and led to colony formation in the co-culture, and these colonies featured with androgen-independent phenotypes [69]. It has been reported that coinjection of tumorigenic rat prostatic fibroblasts enhanced tumor formation of otherwise non-tumorigenic adjacent prostatic epithelial cells via paracrine signaling, leading to the formation of carcinosarcoma [70]. As Hs5 cells utilized herein are tumorigenic whereas HPCa cells are non-tumorigenic in NSG mice (Table 2) but HPCa/Hs5 coinjections initiate serially transplantable tumors, the HPCa/Hs5 tumors, to a certain degree, resemble carcinosarcomas reported earlier [70]. Cell fusion and carcinosarcoma formation can probably help explain why in general the EpCAM^+^ cells in HPCa/Hs5 tumors are rare (i.e., ∼0.2% or less). It is interesting that tumors derived from EpCAM^+^ cells contain both epithelial and mesenchymal-like cells, suggesting that the EpCAM^+^ cells might possess some bi-potential differentiation capacity.

A recent study reported that PCa could be reconstituted from primary HPCa single cells by coinjecting with neonatal mouse mesenchyme under kidney capsule, which significantly increased xenografting rate to 32% compared to 0% without the mesenchyme [43]. It remains unclear whether the reconstituted tumors are serially transplantable. More recently, Domingo-Domenech et al reported that less differentiated HPCa cells marked by low levels of HLA expression and injected in Matrigel can initiate serially transplantable tumors in NSG mice, although the efficiency is rather low, i.e., <10% [71]. This study [71] is fully consistent with ours [25], which, coupled with the present study, strongly suggests that undifferentiated PCa cells are endowed with the unique capability to regenerate PCa in immmunodeficient hosts. Future research should focus on better characterizing the immunophenotypes of undifferentiated PCa cells, which should lead to much improved tumor reconstitution protocols.

## Supporting Information

Figure S1 **Testing antibody specificity in mouse prostate tumors.** Serial sections from the Hi-Myc mouse prostate tumors were stained for HE, Hu-ki67, Hu-mito, or mouse-ki67 antibodies. Both low (i.e., 100x) and high-power (i.e., 400x) magnifications were shown. Note that although mouse-specific Ki-67 antibody stained positively, the human-specific anti-Ki67 and anti-mitochondria antibodies did not manifest any specific staining.
(TIF)

Figure S2 **Histological analysis of Hs5 tumors.** Serial sections were stained for HE, Hu-mito, Hu-ki67, AR, PSA, CK8, CK5 and p63. Both low (i.e., 100x) and high-power (i.e., 400x) magnifications were shown.
(TIF)

Table S1 **Primary antibodies used in the current study.**
(DOC)

Table S2 **Primary tumor (HPCa) samples used in the current study.**
(DOC)

Table S3 **HPCa xenotransplantation using tumor pieces in immunodeficient mice.**
(DOC)

Table S4 **Unsorted or marker-sorted HPCa cells mixed with CAFs fail to initiate transplantable tumors in NOD/SCID mice.**
(DOC)

Table S5 **Unsorted or marker-sorted HPCa cells mixed with Hs5 cells fail to initiate transplantable tumors in NOD/SCID mic.**
(DOC)

Table S6 **Reconstituted ‘prostate’ tumors are independent of Hs5 cells, host, and injection site.**
(DOC)

Table S7 **Cultured Hs5 cells initiate tumor development in NSG mice.**
(DOC)
