# Supplementary figures and images for: Dissociated Primary Human Prostate Cancer Cells Coinjected with the Immortalized Hs5 Bone Marrow Stromal Cells Generate Undifferentiated Tumors in NOD/SCID-γ Mice
Source: PLoS One. 2013 Feb 22;8(2):e56903. doi: 10.1371/journal.pone.0056903 (PMC3579939; doi:10.1371/journal.pone.0056903)

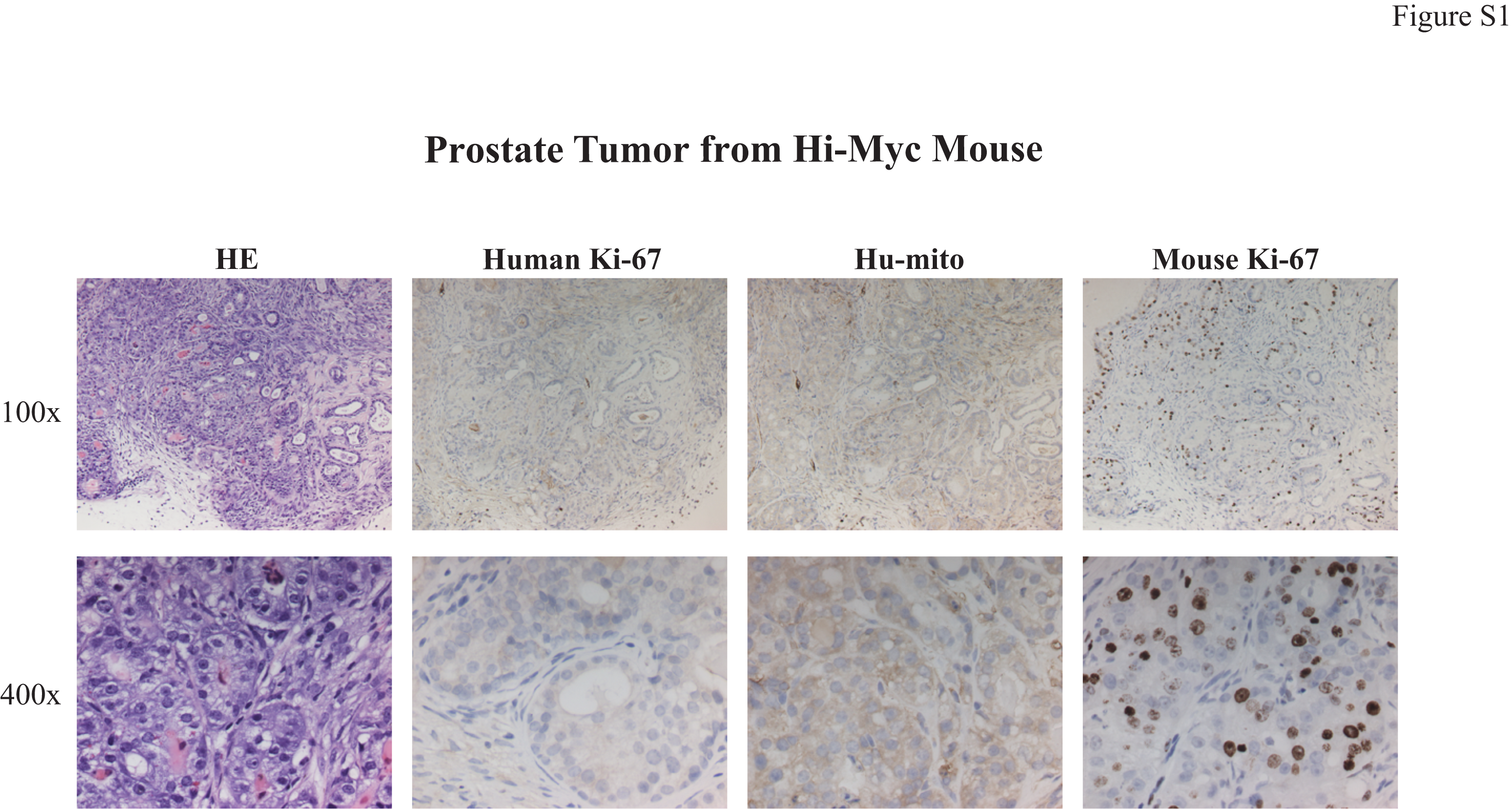

Supplement: Figure S1 — Testing antibody specificity in mouse prostate tumors. Serial sections from the Hi-Myc mouse prostate tumors were stained for HE, Hu-ki67, Hu-mito, or mouse-ki67 antibodies. Both low (i.e., 100x) and high-power (i.e., 400x) magnifications were shown. Note that although mouse-specific Ki-67 antibody stained positively, the human-specific anti-Ki67 and anti-mitochondria antibodies did not manifest any specific staining. (TIF) [file pone.0056903.s001.tif]

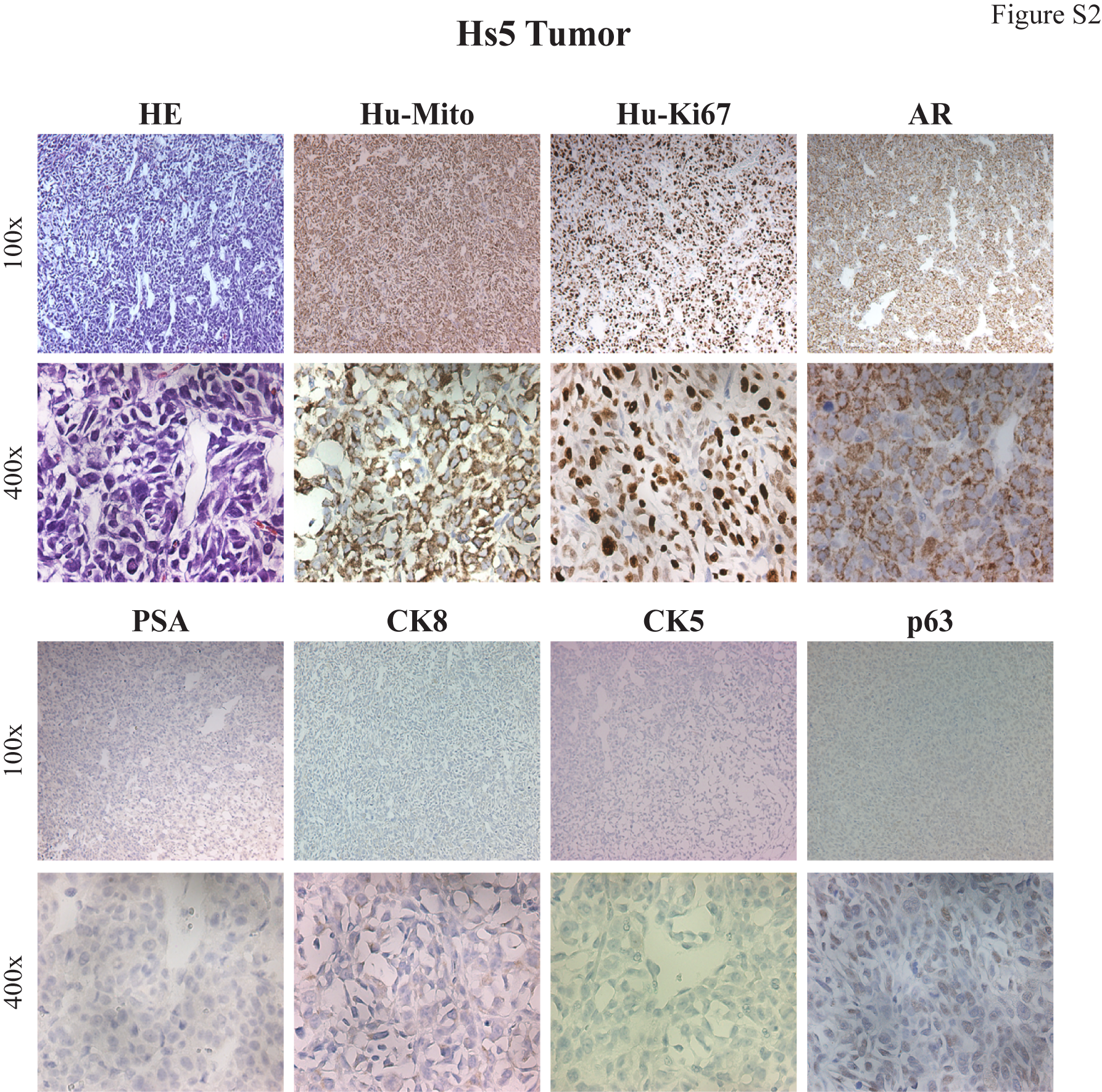

Supplement: Figure S2 — Histological analysis of Hs5 tumors. Serial sections were stained for HE, Hu-mito, Hu-ki67, AR, PSA, CK8, CK5 and p63. Both low (i.e., 100x) and high-power (i.e., 400x) magnifications were shown. (TIF) [file pone.0056903.s002.tif]
