# Supplementary material for: Dissociated Primary Human Prostate Cancer Cells Coinjected with the Immortalized Hs5 Bone Marrow Stromal Cells Generate Undifferentiated Tumors in NOD/SCID-γ Mice
Source: PLoS One. 2013 Feb 22;8(2):e56903. doi: 10.1371/journal.pone.0056903 (PMC3579939; doi:10.1371/journal.pone.0056903)
Supplement: Table S1 — Primary antibodies used in the current study. (DOC) [file pone.0056903.s003.doc]

**Table S1. Primary antibodies** used in the current study

| **Antibody** | **Host/Type** | **Catalog #** | **Dilution** | **Company** | **Usage** | **Remarks** |
| --- | --- | --- | --- | --- | --- | --- |
| AR | mouse monoclonal | sc-7305 | 1:1000 | Santa Cruz | WB | Clone 441 |
| AR | rabbit polyclonal | ab74272 | 1:200 | Abcam | IHC |  |
| PSA | mouse monoclonal | ab2218 | 1:1000 | Abcam | WB | Clone A67-B/E3 |
| PSA | rabbit polyclonal | N1517 | 1:5 | Dako | IHC |  |
| Racemase | mouse monoclonal | sc-81710 | 1:500 | Santa Cruz | WB | Clone 2A10F3 |
| Racemase | rabbit monoclonal | M3616 | 1:300 | Dako | IHC | Clone 13H4 |
| p63 | mouse monoclonal | #559951 | 1:1000 | BD Pharmingen | WB | Clone 4A4 |
| p63 | mouse monoclonal | sc-8431 | 1:100 | Santa Cruz | IHC | Clone 4A4 |
| CK5 | rabbit polyclonal | #PRB-160P | 1:500 | Covance | IHC |  |
| CK8 | rabbit polyclonal | Troma-1 | 1:10 | DSHB | IHC |  |
| CK18 | rabbit polyclonal | sc-28264 | 1:500 | Santa Cruz | WB |  |
| β-actin | rabbit polyclonal | #4967 | 1:1000 | Cell Signaling | WB |  |
| GAPDH | rabbit polyclonal | ab9485 | 1:2000 | Abcam | WB |  |
| Ki-67 | mouse monoclonal | M7240 | 1:300 | Dako | IHC | Clone MIB-1, human specific |
| Ki-67 | rat monoclonal | M7249 | 1:200 | Dako | IHC | Clone TEC-3, mouse specific |
| mitochondria | mouse monoclonal | MAB1273 | 1:2000 | Millipore | IHC | Clone 113-1, human specific |
| EpCAM | mouse monoclonal | 130061101 | 1:5 | Miltenyi Biotec | MACS | Microbeads conjugated |
| EpCAM | mouse monoclonal | 469326 | 1:20 | eBioscience | FACS | PerCP-eFluor 710 conjugated |
| H-2K[d] | mouse monoclonal | 553564 | 1:5 | BD Pharmingen | FACS | Clone SF1-1.1, Biotinlyated |
| CD44 | mouse monoclonal | 555478 | 1:10 | BD Pharmingen | FACS | FITC conjugated |

Abbreviations used: AR, androgen receptor; PSA, prostate specific antigen; CK5, cytokeratin 5, CK8, cytokeratin 8; EpCAM, epithelial cell adhesion molecule; IHC, immunohistochemistry; WB, western blotting; MACS, magnetic-activated cell sorting; FACS, fluorescence-activated cell sorting.
