# Supplementary material for: Dissociated Primary Human Prostate Cancer Cells Coinjected with the Immortalized Hs5 Bone Marrow Stromal Cells Generate Undifferentiated Tumors in NOD/SCID-γ Mice
Source: PLoS One. 2013 Feb 22;8(2):e56903. doi: 10.1371/journal.pone.0056903 (PMC3579939; doi:10.1371/journal.pone.0056903)
Supplement: Table S2 — Primary tumor (HPCa) samples used in the current study. (DOC) [file pone.0056903.s004.doc]

**Table S2. Primary tumor (HPCa) samples** used in the current study

| **Patient** | **Age** | **Gleason** | **Experiments** |
| --- | --- | --- | --- |
| HPCa2 | 73 | 6 (3+3) | cell injection |
| HPCa3 | 73 | 7 (3+4) | cell injection, tumor piece implant |
| HPCa4 | 63 | 7 (4+3) | cell injection, tumor piece implant |
| HPCa5 | 72 | 9 (4+5) | cell injection, tumor piece implant |
| HPCa6 | 68 | 7 (3+4) | tumor piece implant |
| HPCa7 | 66 | 7 (4+3) | cell injection, tumor piece implant |
| HPCa8 | 56 | 6 (3+3) | cell injection, tumor piece implant |
| HPCa9 | 62 | 6 (3+3) | cell injection, tumor piece implant |
| HPCa10 | 71 | 6 (3+3) | cell injection, tumor piece implant |
| HPCa11 | 69 | 7 (4+3) | cell injection, tumor piece implant |
| HPCa12 | 59 | 7 (4+3) | cell injection, tumor piece implant |
| HPCa13 | 62 | 6 (3+3) | cell injection, tumor piece implant |
| HPCa14 | 68 | 7 (4+3) | cell injection, tumor piece implant |
| HPCa15 | 64 | 8 (4+4) | cell injection, tumor piece implant |
| HPCa16 | 58 | 6 (3+3) | cell injection, tumor piece implant |
| HPCa17 | 60 | 7 (4+3) | tumor piece implant |
| HPCa18 | 68 | 7 (4+3) | cell injection, tumor piece implant |
| HPCa19 | 61 | 9 (4+5) | tumor piece implant |
| HPCa20 | 63 | 9 (4+5) | tumor piece implant |
| HPCa21 | 60 | 10 (5+5) | cell injection, tumor piece implant |
| HPCa22 | 63 | 7 (3+4) | tumor piece implant |
| HPCa23 | 72 | 7 (3+4) | tumor piece implant |
| HPCa24 | 62 | 9 (4+5) | tumor piece implant |
| HPCa25 | 58 | 8 (3+5) | tumor piece implant |
| HPCa26 | 60 | 6 (3+3) | tumor piece implant |
| HPCa27 | 47 | 8 (4+4) | cell injection, tumor piece implant |
| HPCa28 | 51 | 9 (5+4) | tumor piece implant |
| HPCa32 | 55 | 7 (3+4) | tumor piece implant |
| HPCa33 | 58 | 7 (3+4) | tumor piece implant |
| HPCa34 | 58 | 7 (4+3) | cell injection |
| HPCa36 | 65 | 9 (4+5) | tumor piece implant |
| HPCa37 | 59 | 9 (4+5) | cell injection, tumor piece implant |
| HPCa39 | 63 | 7 (3+4) | cell injection, tumor piece implant |
| HPCa40 | 58 | 7 (3+4) | tumor piece implant |
| HPCa42 | 54 | 9 (5+4) | cell injection, tumor piece implant |
| HPCa43 | 55 | 7 (4+3) | tumor piece implant |
| HPCa44 | 61 | 9 (5+4) | tumor piece implant |
| HPCa45 | 70 | 9 (4+5) | cell injection, tumor piece implant |
| HPCa46 | 51 | 9 (4+5) | tumor piece implant |
| HPCa48 | 59 | 7 (3+4) | tumor piece implant |
| HPCa49 | 57 | 7 (3+4) | tumor piece implant |
| HPCa50 | 60 | 7 (4+3) | tumor piece implant |
| HPCa51 | 44 | 7 (4+3) | tumor piece implant |
| HPCa52 | 63 | 8 (4+4) | cell injection, tumor piece implant |
| HPCa53 | 58 | 7 (4+3) | tumor piece implant |
| HPCa54 | 61 | 8 (4+4) | tumor piece implant |
| HPCa55 | 56 | 7 (3+4) | tumor piece implant |
| HPCa56 | 52 | 7 (3+4) | tumor piece implant |
| HPCa57 | 53 | 7 (3+4) | IHC, WB, RT-PCR, cytogenetic analysis, cell injection, tumor piece implant |
| HPCa58 | 58 | 7 (3+4) | IHC, WB, RT-PCR, cell injection, tumor piece implant |
| HPCa69 | 74 | 8 (4+4) | cell injection, tumor piece implant |
| HPCa70 | 60 | 7 (3+4) | IHC, WB, RT-PCR, cytogenetic analysis, cell injection, tumor piece implant |
| HPCa71 | 64 | 6 (3+3) | cell injection |
| HPCa74 | 59 | 7 (3+4) | tumor piece implant |
| HPCa75 | 76 | 8 (4+4) | cell injection |
| HPCa76 | 64 | 7 (4+3) | tumor piece implant |
| HPCa79 | 67 | 7 (4+3) | tumor piece implant |
| HPCa80 | 65 | 9 (4+5) | WB, RT-PCR, cell injection, tumor piece implant |
| HPCa82 | 54 | 6 (3+3) | cell injection, tumor piece implant |
| HPCa83 | 69 | 7 (3+4) | WB, RT-PCR, cell injection, tumor piece implant |
| HPCa84 | 66 | 7 (4+3) | WB, RT-PCR, cell injection, tumor piece implant |
| HPCa85 | 62 | 7 (3+4) | WB, RT-PCR, cell injection |
| HPCa86 | 57 | 7 (4+3) | tumor piece implant |
| HPCa87 | 57 | 9 (4+5) | IHC, WB, RT-PCR, cytogenetic analysis, cell injection, tumor piece implant |
| HPCa88 | 72 | 9 (5+4) | tumor piece implant |
| HPCa89 | 55 | 9 (4+5) | cell injection, tumor piece implant |
| HPCa90 | 66 | 8 (4+4) | tumor piece implant |
| HPCa91 | 60 | 8 (3+5) | WB, RT-PCR, cell injection, tumor piece implant |
| HPCa92 | 57 | 7 (4+3) | WB, RT-PCR, cell injection, tumor piece implant |
| HPCa93 | 58 | 7 (3+4) | cell injection |
| HPCa96 | 55 | 9 (5+4) | WB, cell injection, tumor piece implant |
| HPCa100 | 54 | 7 (3+4) | tumor piece implant |
| HPCa101 | 71 | 9 (4+5) | IHC, WB, RT-PCR, flow analysis, cell injection, tumor piece implant |
| HPCa103 | 74 | 7 (3+4) | tumor piece implant |
| HPCa104 | 54 | 7 (3+4) | tumor piece implant |
| HPCa105 | 63 | 9 (4+5) | tumor piece implant |
| HPCa 114 | 59 | 9 (5+4) | tumor piece implant |

Abbreviations used: HPCa, primary human prostate cancer samples; IHC, immunohistochemistry; RT-PCR, reverse transcription – polymerase chain reaction; WB, western blotting.
