# Supplementary material for: Dissociated Primary Human Prostate Cancer Cells Coinjected with the Immortalized Hs5 Bone Marrow Stromal Cells Generate Undifferentiated Tumors in NOD/SCID-γ Mice
Source: PLoS One. 2013 Feb 22;8(2):e56903. doi: 10.1371/journal.pone.0056903 (PMC3579939; doi:10.1371/journal.pone.0056903)
Supplement: Table S3 — HPCa xenotransplantation using tumor pieces in immunodeficient mice. (DOC) [file pone.0056903.s005.doc]

**Table S3. HPCa xenotransplantation using tumor pieces in immunodeficient mice***

| **Patient (Gleason)** | **Harvest time**  **(days)** | **Host** | **Tumor take** | | |
| --- | --- | --- | --- | --- | --- |
|  |  |  | s.c | KC | AP |
| HPCa8 (GS6) | 146 | N/S | - | 0/4 | 0/1 |
| HPCa10 (GS6) | 152 | N/S | - | 0/5 | 0/1 |
| HPCa13 (GS6) | 154 | N/S | 0/2 | 0/6 | - |
| HPCa16 (GS6) | 168 | N/S | - | 0/7 | 0/7 |
| HPCa26 (GS6) | 158 | N/S | 8/8 | 2/4 | 2/2 |
| HPCa82 (GS6) | 273 | N/S | 0/6 | - | - |
| HPCa4 (GS7) | 121 | N/S | - | 0/8 | - |
| HPCa6 (GS7) | 184 | N/S | - | 0/3 | 0/4 |
| HPCa7 (GS7) | 181 | N/S | - | 0/3 | 0/2 |
| HPCa9 (GS7) | 167 | N/S | - | - | 0/1 |
| HPCa11 (GS7) | 161 | N/S | 0/4 | - | - |
| HPCa12 (GS7) | 154 | N/S | 0/2 | 0/6 | - |
| HPCa14 (GS7) | 181 | N/S | 4/4 | 0/5 | 1/4 |
| HPCa17 (GS7) | 183 | N/S | 0/4 | 0/4 | 0/2 |
| HPCa18 (GS7) | 154 | N/S | 2/4 | 0/2 | 0/3 |
| HPCa22 (GS7) | 95 | N/S | 1/6 | - | 0/4 |
| HPCa23 (GS7) | 91 | N/S | 3/6 | 17/22 | - |
| HPCa32 (GS7) | 95 | N/S | 2/2 | - | - |
| HPCa33 (GS7) | 81 | N/S | 1/2 | - | - |
| HPCa34 (GS7) | 123 | N/S | 4/4 | - | - |
| HPCa39 (GS7) | 94 | N/S | 5/6 | - | - |
| HPCa40 (GS7) | 171 | N/S | 6/8 | - | - |
| HPCa43 (GS7) | 145 | N/S | 2/2 | - | - |
| HPCa48 (GS7) | 182 | N/S | - | 3/6 | - |
| HPCa49 (GS7) | 184 | N/S | 2/8 | - | - |
| HPCa50 (GS7) | 116 | N/S | 0/7 | - | - |
| HPCa51 (GS7) | 163 | N/S | 6/6 | - | - |
| HPCa53 (GS7) | 167 | N/S | 2/6 | - | - |
| HPCa55 (GS7) | 139 | N/S | 6/6 | - | - |
| HPCa56 (GS7) | 132 | N/S | 3/3 | - | - |
| HPCa57 (GS7) | 154 | N/S | 4/6 | - | - |
| HPCa58 (GS7) | 154 | N/S | 6/6 | - | - |
| HPCa70 (GS7) | 160 | N/S | 4/6 | - | - |
| HPCa83 (GS7) | 185 | N/S | 1/3 | - | - |
| HPCa86 (GS7) | 270 | N/S | 4/4 | - | - |
| HPCa32 (GS7) | 95 | Rag2 | 3/4 | - | - |
| HPCa33 (GS7) | 81 | Rag2 | 3/4 | - | - |
| HPCa34 (GS7) | 123 | Rag2 | 8/8 | - | - |
| HPCa74 (GS7) | 158 | Rag2 | 0/6 | - | - |
| HPCa76 (GS7) | 299 | NSG | 1/2 | - | - |
| HPCa79 (GS7) | 265 | NSG | 3/4 | - | - |
| HPCa83 (GS7) | 185 | NSG | 2/3 | - | - |
| HPCa84 (GS7) | 279 | NSG | 4/6 | - | - |
| HPCa86 (GS7) | 270 | NSG | 2/4 | - | - |
| HPCa92 (GS7) | 149 | NSG | 4/6 | - | - |
| HPCa100 (GS7) | 271 | NSG | 5/5 | - | - |
| HPCa103 (GS7) | 276 | NSG | 6/6 | - | - |
| HPCa104 (GS7) | 275 | NSG | 4/6 | - | - |
| HPCa15 (GS8) | 130 | N/S | 4/6 | 1/9 | 0/4 |
| HPCa25 (GS8) | 125 | N/S | 6/6 | - | - |
| HPCa27 (GS8) | 144 | N/S | 8/8 | 5/6 | 3/10 |
| HPCa52 (GS8) | 173 | N/S | 5/6 | - | - |
| HPCa54 (GS8) | 140 | N/S | 9/10 | - | - |
| HPCa69 (GS8) | 178 | N/S | 4/4 | - | - |
| HPCa25 (GS8) | 125 | Rag2 | 3/4 | 2/2 | - |
| HPCa69 (GS8) | 178 | Rag2 | 2/4 | - | - |
| HPCa90 (GS8) | 252 | NSG | 2/3 | - | - |
| HPCa91 (GS8) | 244 | NSG | 5/6 | - | - |
| HPCa5 (GS9) | 40 | N/S | - | 1/2 | - |
| HPCa19 (GS9) | 153 | N/S | 4/4 | - | 2/2 |
| HPCa20 (GS9) | 145 | N/S | 2/2 | 1/3 | 1/2 |
| HPCa24 (GS9) | 88 | N/S | 5/5 | - | - |
| HPCa28 (GS9) | 172 | N/S | 2/4 | 3/3 | 1/2 |
| HPCa36 (GS9) | 159 | N/S | 6/6 | - | - |
| HPCa37 (GS9) | 173 | N/S | 6/6 | - | - |
| HPCa42 (GS9) | 129 | N/S | 2/4 | - | - |
| HPCa44 (GS9) | 173 | N/S | 8/10 | - | - |
| HPCa45 (GS9) | 154 | N/S | 6/6 | - | - |
| HPCa46 (GS9) | 188 | N/S | 3/4 | - | - |
| HPCa21 (GS9) | 124 | N/S | 1/6 | 1/4 | 0/4 |
| HPCa80 (GS9) | 258 | Rag2 | 1/2 | - | - |
| HPCa87 (GS9) | 269 | NSG | 5/8 | - | - |
| HPCa88 (GS9) | 253 | NSG | 4/4 | - | - |
| HPCa89 (GS9) | 249 | NSG | 5/6 | - | - |
| HPCa96 (GS9) | 257 | NSG | 6/6 | - | - |
| HPCa101 (GS9) | 101 | NSG | 1/4 | - | - |
| HPCa105 (GS9) | 256 | NSG | 2/3 | - | - |
| HPCa114 (GS9) | 274 | NSG | 6/6 | - | - |

*HPCa pieces (~2-3 mm^3^) were implanted in the indicated strains of immunodeficient male mice supplemented with

testosterone pellets. For s.c experiments, tumor pieces soaked in 50% Matrigel (MG) were surgically implanted.

For KC experiments, tumor pieces were directly implanted in the kidney capsule of the host. For AP implantation

experiments, tumor pieces were surgically grafted in the AP tubules. “-“, not done.
