# Supplementary material for: Dissociated Primary Human Prostate Cancer Cells Coinjected with the Immortalized Hs5 Bone Marrow Stromal Cells Generate Undifferentiated Tumors in NOD/SCID-γ Mice
Source: PLoS One. 2013 Feb 22;8(2):e56903. doi: 10.1371/journal.pone.0056903 (PMC3579939; doi:10.1371/journal.pone.0056903)
Supplement: Table S4 — Unsorted or marker-sorted HPCa cells mixed with CAFs fail to initiate transplantable tumors in NOD/SCID mice. (DOC) [file pone.0056903.s006.doc]

**Table S4**. Unsorted or marker-sorted HPCa cells mixed with CAFs fail to initiate transplantable tumors in NOD/SCID mice

| **HPCa sample** | **Marker/cell number** | **Site/Harvest time (d)** | **Incidence** |
| --- | --- | --- | --- |
| HPCa71 (GS6) | Unsorted/250k (1x), 500k (1x), 1000k (1x) | s.c (180) | 0/3 |
| HPCa34 (GS7) - 1^o^ xenograft | CD44+/1k (3x), 10k (3x), 50k (1x) | s.c (139) | 0/7 |
|  | CD44-/1k (2x), 10k (2x), 50k (3x), 500k (1x) | s.c (139) | 0/8 |
| HPCa39 (GS7) - 1^o^ xenograft | CD44+/1k (8x), 10k (8x), 100k (2x) | s.c (147) | 1/4 |
|  | CD44-/1k (6x), 10k (6x), 100k (6x) | s.c (147) | 2/6 |
| HPCa52 (GS8) | CD44+/100k (1x) | s.c (163) | 0/1 |
|  | CD44-/100k (2x) | s.c (163) | 0/2 |
|  | Unsorted /500k (1x) | s.c (163) | 0/1 |
| HPCa27 (GS8) - 1^o^ xenograft | CD44+/1k (5x), 10k (4x), 50k (1x) | s.c (150) | 0/10 |
|  | CD44-/1k (2x), 10k (2x), 50k (2x), 100k (2x) | s.c (150) | 0/8 |
| HPCa45 (GS9) - 1^o^ xenograft | CD44+/1k (2x), 50k (1x) | KC (113) | 0/3 |
|  | CD44-/50k (1x) | KC (113) | 0/1 |
|  | Unsorted /300k (2x) | KC (113) | 0/2 |

**Total: 3/56 = 5.4%**

For s.c injections, cells were mixed with 100,000 carcinoma-associated fibroblasts (CAFs) and implanted subcutaneously in 50% Matrigel; For KC transplantations, cells were first recombined with 250,000 CAFs and then transplanted under the kidney capsule. All injections/transplantations were carried out in 6-8 week old male NOD/SCID mice supplemented with testosterone pellet.
