# Supplementary material for: Dissociated Primary Human Prostate Cancer Cells Coinjected with the Immortalized Hs5 Bone Marrow Stromal Cells Generate Undifferentiated Tumors in NOD/SCID-γ Mice
Source: PLoS One. 2013 Feb 22;8(2):e56903. doi: 10.1371/journal.pone.0056903 (PMC3579939; doi:10.1371/journal.pone.0056903)
Supplement: Table S5 — Unsorted or marker-sorted HPCa cells mixed with Hs5 cells fail to initiate transplantable tumors in NOD/SCID mic. (DOC) [file pone.0056903.s007.doc]

**Table S5. Unsorted or marker-sorted HPCa cells mixed with Hs5 cells fail to initiate transplantable tumors in NOD/SCID mice***

| HPCa sample | Marker/cell number | Harvest time (d) | Incidence |
| --- | --- | --- | --- |
| HPCa71 (GS6) | Unsorted/250k (2x), 500k (2x), 1M (2x) | 131 | 0/6 |
| HPCa82 (GS6) | Unsorted/10k (2x), 100k (1x) | 273 | 0/3 |
| HPCa70 (GS7) | CD44^+^/10k (6x) | 175 | 0/6 |
|  | CD44^-^/10k (6x), 100k (2x) | 175 | 0/8 |
| HPCa83 (GS7 | Unsorted/10k (5x) | 185 | 0/5 |
| HPCa93 (GS7) | Unsorted/1k (2x), 10k (2x), 40k (2x) | 261 | 1/6 |
| HPCa52 (GS8) | Unsorted/500k (1x) | 163 | 0/1 |
|  | CD44^+^/100k (1x) | 163 | 1/1 |
|  | CD44^-^/100k (2x) | 163 | 2/2 |
| HPCa69 (GS8) | Unsorted/960k (1x) | 178 | 0/1 |
| HPCa75 (GS8) | Unsorted/25k (1x) | 203 | 0/1 |
| HPCa91 (GS8) | Unsorted/100 (1x), 1k (1x), 10k (1x), 50k (1x) | 123 | 0/4 |
| HPCa87 (GS9) | Unsorted/1k (4x), 10k (4x), 100k (2x) | 272 | 0/10 |

**Total: 4/54 = 7.4%**

*Unsorted or purified CD44^+^/CD44^-^ HPCa cells were mixed with 100,000 Hs5 cells and injected subcutaneously in 50% Matrigel in 6-8 weeks old male NOD/SCID mice supplemented with testosterone pellet.
