# Supplementary material for: Dissociated Primary Human Prostate Cancer Cells Coinjected with the Immortalized Hs5 Bone Marrow Stromal Cells Generate Undifferentiated Tumors in NOD/SCID-γ Mice
Source: PLoS One. 2013 Feb 22;8(2):e56903. doi: 10.1371/journal.pone.0056903 (PMC3579939; doi:10.1371/journal.pone.0056903)
Supplement: Table S6 — Reconstituted ‘prostate’ tumors are independent of Hs5 cells, host, and injection site. (DOC) [file pone.0056903.s008.doc]

**Table S6. Reconstituted ‘prostate’ tumors are independent of Hs5 cells, host, and injection site***

| HPCa sample | Marker/Cell number | Co-injection | Injection site/ Precondition/Host^#^ | Harvest time (days) | Incidence^c^ |
| --- | --- | --- | --- | --- | --- |
| HPCa57 (GS7)-2^o^ | Unsorted/200k (2x), 500k (2x), 1M (2x) | 100k Hs5 | s.c/NSG | 31 | 3/3 |
|  | CD44^+^/1k (3x), 100k (6x) | 100k Hs5 | s.c/NSG | 35 | 5/6 |
|  | CD44^-^/1k (3x), 100k (3x), 1M(2x) | 100k Hs5 | s.c/NSG | 35 | 5/6 |
| HPCa57 (GS7)-3^o^ | CD44^+^/1k (4x), 5k (4x), 10k(2x), 50k (2x) | 100k Hs5 | s.c/NSG | 77 | 8/8 |
|  | CD44^-^/1k (7x), 10k (1x) | 100k Hs5 | s.c/NSG | 77 | 4/8 |
| HPCa57 (GS7)-4^o^ | Unsorted/10k (6x), 100k (5x) | 100k Hs5 | s.c/NSG | 56 | 8/9 |
|  | Unsorted/10k (6x), 100k (4x) | - | s.c/NSG | 56 | 3/8 |
| HPCa57 (GS7)-5^o^ | Unsorted/1k (2x), 10k (2x), 100k (2x) | - | s.c/NSG | 37 | 6/6 |
|  | CD44^+^/100 (2x), 1k (2x), 10k (2x), 100k (2x) | - | s.c/NSG | 37 | 8/8 |
|  | CD44^-^/1k (2x), 10k (2x), 100k (2x) | - | s.c/NSG | 37 | 6/6 |
| HPCa80 (GS9)-1^o^ | Unsorted/400k(4x) | 100k Hs5 | DP/NSG | 35 | 4/4 |
|  | Unsorted/400k(2x) | - | DP/NSG | 35 | 1/2 |
| HPCa80 (GS9)-2^o^ | Unsorted/300k(2x) | - | DP/NSG | 50 | 2/2 |
|  | Unsorted/400k(3x) | - | DP/NSG | 50 | 2/3 |
| HPCa70 (GS7)-2^o^ | Unsorted/1M (2x), 500k (4x), 100k (4x) | 100k Hs5 | s.c/NS | 63 | 8/10 |
| HPCa70 (GS7)-3^o^ | Unsorted/500k(5x) | 100k Hs5 | s.c/NS | 33 | 5/5 |
|  | Unsorted/500k (5x) | - | s.c/NS | 33 | 5/5 |
| HPCa70 (GS7)-4^o^ | Unsorted/100k (5x) | - | s.c/NS | 46 | 5/5 |

*Bulk or CD44^+^/CD44^-^ HPCa cells were purified from primary (1º) xenografts and used in subsequent (2º, 3º, etc) xenotransplantations (either s.c or in the DP) in the presence or absence (-) of 100,000 Hs5 cells in male NOD/SCID (NS) or NSG mice supplemented with testosterone pellet.
