# Supplementary material for: Dissociated Primary Human Prostate Cancer Cells Coinjected with the Immortalized Hs5 Bone Marrow Stromal Cells Generate Undifferentiated Tumors in NOD/SCID-γ Mice
Source: PLoS One. 2013 Feb 22;8(2):e56903. doi: 10.1371/journal.pone.0056903 (PMC3579939; doi:10.1371/journal.pone.0056903)
Supplement: Table S7 — Cultured Hs5 cells initiate tumor development in NSG mice. (DOC) [file pone.0056903.s009.doc]

**Table S7. Cultured Hs5 cells initiate tumor development in NSG mice***

| **Cell number** | **Harvest Time (days)** | **Incidence^b^** |
| --- | --- | --- |
| 100k (2x) | 232 | 1/2 |
| 100k (2x) | 34 | 0/2^#^ |
| 100k (2x) | 153 | 1/2 |
| 100k (1x) | 180 | 1/1 |
| 100k (2x) | 42 | 0/2^#^ |
| 100k (2x) | 150 | 1/2 |
| 100k (2x) | 197 | 2/2 |
| 100k (2x) | 140 | 2/2 |
| 100k (4x) | 139 | 4/4 |
| 100k (2x) | 104 | 1/2 |
| 100k (2x) | 149 | 0/2 |
| 100k (2x) | 133 | 1/2 |
| 50k (2x), 10k (2x), 1k (2x) | 232 | 2/6 |
| 500k (6x), 100k (6x), 10k (6x), 1k (6x), 100 (6x) | 147 | 16/30 |

**Total: 32/61 = 52.5%**

*Hs5 cells were subcutaneously injected in 50% Matrigel into 6-8 week old NSG mice supplemented with testosterone pellet.

# Mice died on days 34 and 42 respectively, and no tumor was detected.
